# Diterpenoids from *Euphorbia gedrosiaca* as Potential Anti-Proliferative Agents against Breast Cancer Cells

**DOI:** 10.3390/metabo13020225

**Published:** 2023-02-03

**Authors:** Zeinab Yazdiniapour, Mohammad Hossein Sohrabi, Newsha Motinia, Behzad Zolfaghari, Pegah Mehdifar, Mustafa Ghanadian, Virginia Lanzotti

**Affiliations:** 1 Department of Pharmacognosy, Isfahan University of Medical Sciences, Isfahan 81746-73461, Iran; 2 Dipartimento di Agraria, Università di Napoli Federico II, 80055 Portici, Napoli, Italy

**Keywords:** *Euphorbia gedrosiaca*, premyrsinane diterpenoids, MDA-MB-231, MCF-7, cytotoxic activity

## Abstract

Isolated diterpenes from various species of *Euphorbia* are important compounds for drug discovery with a broad spectrum of structures and biological effects. In this study, *Euphorbia gedrosiaca*, one of the endemic species of Iran, was analyzed in terms of the presence and structural determination of diterpenoid compounds. They were extracted with dichloromethane/acetone (2:1) from aerial parts of this plant and purified by chromatographic methods such as MPLC and HPLC. Four premyrsinane compounds and one myrsinane diterpene were isolated from *Euphorbia gedrosiaca*. They were characterized by extensive 1D and 2D NMR and HRMS analyses. Additionally, their activities were evaluated against two breast cancer cell lines, MDA-MB-231 and MCF-7, by MTT proliferation assay. They exhibited cytotoxic effects in a dose-dependent manner with promising results, which can help to find possible therapeutic application of diterpenoids in breast cancer treatment.

## 1. Introduction

**Figure 1:**
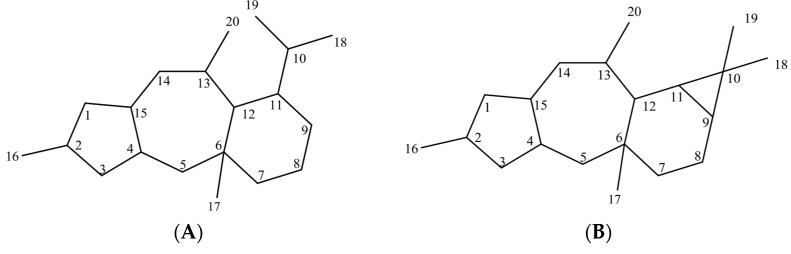
(**A**) Skeletal structure of myrsinanes; (**B**) Skeletal structure of premyrsinanes.

As the main genera of the Euphorbiaceae family, *Euphorbia* has more than 2000 species that grow in all temperate and tropical regions. They cover a wide range of habitats and are distinguished by their particular inflorescences, designated as Cyathia [1,2]. Medicinal species are traditionally used to treat warts, and as a paste to relieve pains [3]. From 2013 to 2019, 455 diterpenoids were isolated from 53 species of *Euphorbia* [4], most of which are effective in a broad range of biological activities with potential usage in health maintenance [5]. Cyclomyrsinane diterpenoids with 5/7/6/3-tetracyclic carbon framework, myrsinane with 5/7/6 tricyclic ring system (Figure 1A), and premyrsinane diterpenoids with 5/7/6/3-tetracyclic carbon (Figure 1B) belong to myrsinane-type diterpenoids [4]. Premyrsinane diterpenoids are obtained by the cyclization at C-12 and C-6 of lathyrane diterpenoids [4] and might be rearranged to myrsinane structures by breaking of C-9 and C-10 bond [4].

They have chemotaxonomic significance and were isolated only from *Euphorbia* species, including *E. prolifera* [6,7], *E. sogdiana* [8], *E. dracunculoides* [9,10], *E. lathyris* [11] *E. sanctae-catharinae* [12], *E. macroclada* [13], *E. falcata* [14,15], and *E. aleppica* [16] with antifungal effects against plant pathogenic fungi, cytotoxic activities, and modulating the drug resistance in cancer cells [7,8,15]

*E. gedrosiaca*, also identified by the synonym *Tithymalus gedrosiacus,* is closely related to *E. erythradenia*. It is a rare localized endemic plant in the Iranian plateau that grows mainly in the center and southeastern parts of Iran [17,18]. In the previous research on this plant, two new myrsinane and two known cyclomyrsinane-type diterpenes with moderate cytotoxicity and proapoptotic properties against B16F10 and A375 melanoma skin tumor cells were reported. In the cycle arrest analysis, a significant increase was reported in the G2/M phases for both myrsinane and cyclomyrsinane compounds [19]. In this paper, our investigation led to the isolation of four premyrsinane diterpenes and one myrsinane diterpene from this plant, along with the determination of their cytotoxic activities against MCF-7 and MDA-MB 231 breast cancer cells by standard MTT assay.

## 2. Materials and Methods

### 2.1. General Experimental Procedures

^1^H and ^13^C NMR spectra were recorded on Bruker 400 (^1^H at 400 MHz and ^13^C at 100 MHz) spectrometer (Bruker Corporation, Billerica, MA, USA) using CDCl_3_ (CDCl_3_: δ_H_ 7.26, δ_C_ 77.16) as a solvent. Mass spectra were recorded on an Agilent 1100 SL series LC mass spectrometer (Agilent Technologies, Santa Clara, CA, USA). Medium-pressure liquid chromatography (MPLC) was performed on Büchi 861 equipment (BÜCHI Labortechnik AG, Flawil, Switzerland) using silica gel (15–40 mesh, Merck) as the stationary phase. HPLC was performed in isocratic mode on a Waters 515 equipment with a refractive index detector (Waters 2414) and Dual λ Absorbance Detector (Waters 2487), using semipreparative YMC-Pack Sil 10 µm (20 × 250 mm i.d) and Waters Spherisorb 5 µm (10 × 250 mm i.d.) columns (Waters Corp, Milford, MA, USA). Thin-layer chromatography (TLC) was performed on Merck TLC silica gel plates (Merck & Co., Inc., Rahway, NJ, USA) with hexane-acetone (7:3) as a mobile phase and cerium sulfate in 2N sulfuric acid as a reagent for visualizing the spots.

### 2.2. Plant Materials

Aerial parts of *E. gedrosiaca* Rech.f., Aellen and Esfand., were collected from the South of Nehbandan, South Khorasan province (Iran) in May 2013 in its flowering time. It was identified by Amir Hossein Pahlevan, Department of Botany, Herbaceous Sciences Research Center at the Ferdowsi University of Mashhad, and a voucher specimen (Sam-3638) was kept at the Samsam-Shariat herbarium, Department of Pharmacognosy, Isfahan University of Medical Sciences, Iran.

### 2.3. Extraction Procedure

Plant material was air-dried in the shade (4 kg), and extracted with dichloromethane: acetone (2:1 *v*/*v*) to obtain 170 g of deep green crude extract. It was filtered on a C-18 cartridge using MeOH: H2O (6:4) to get a brown color extract free from unwanted chlorophylls, and fatty contents. The fraction eluted by MeOH: H_2_O (6:4 *v*/*v*, 57.1 g) was concentrated and resubmitted on MPLC silica gel (25–40 μm) column (49 × 46 mm) with hexane: EtOAc (1: 97:3; 2: 95:5; 3: 90:10; 4: 85:15; 5: 80:20; 6: 75:25; 7: 70:30; 8: 60:40). Based on a preliminary H-NMR, fractions 5-7 with characteristic peaks of diterpene polyesters were added together (7g), and chromatographed on silica gel MPLC column (15–25 μm; 26 × 460) with a linear gradient solvent system hexane: EtOAc (5 to 50%). Fr. 5 (3870 mg) eluted in hexane: EtOAc (8:2) was purified with hexane: EtOAc (85:15) and afforded **1** (1.4 mg), **2** (40 mg), and **3** (2.6 mg). Fr.9 (3.02 g) eluted in hexane: EtOAc (70:30) was purified on the same MPLC column using hexane: EtOAc (80:20) resulting in **5** (4.1 mg). Fr. 10 (2 g), eluted with 40% EtOAc in hexane, was chromatographed on a silica gel MPLC column (15–25 μm; 26 × 460) with hexane: EtOAc (10% to 35%). Fr.1 (70 mg) was further purified with hexane: EtOAc (90:10) to yield **4** (2.5 mg). To see HPLC chromatograms, refer to the Supplementary Materials, Figures S35–S37.

### 2.4. MTT Viability Assay

MDA MB-231 and MCF-7 (ATCCR HTB-26 and ATCCR HTB-22) breast cancer cells, originated from Pasteur Institute (IPI, Tehran, I.R. Iran), were cultured in RPMI1640 with streptomycin, penicillin, and fetal bovine serum (FBS) (100 µg/mL, 100 units/mL, 10%) with criteria of 5% carbon dioxide, 37 °C, and 95% humidity for a few days. Mouse normal fibroblast cell line (NCTC clone 929 from the same institute) was cultured in DMEM+10% FBS+ 1% penicillin/streptomycin in the same conditions. After incubation, cells were seeded at 7 × 10^3^ cells in each well of ninety-six well plates for 12 h. Then, the media were changed, and sample compounds in concentrations of 1, 10, and 100 µM were added. For L929 normal cell line, samples were added in the concentrations of 1, 10, 100, 250, and 500 µM. Placebo with the same amount of DMSO in each concentration used for compounds and Taxol in concentrations of 0.001, 0.01, 0.1, 1, and 10 µM were added as the negative and positive controls. After 24 h incubation time, the reduction agent (MTT, 5 mg/mL, 20 µL) was added to each well and incubated for 4 h to let the MTT be reduced into formazan derivative by the mitochondria of survived cells. The media was then changed with DMSO (200 µL) to solubilize the insoluble formazan crystals, and the absorbance was read by a Synergy ELISA reader (BioTek Instruments, Winooski, VT, USA) at 570 nm [20].

## 3. Results

### 3.1. Spectroscopic Data of the Isolated Compounds

1. 13β-O-propanoyl-5α-O-methylbutanoyl-7α,13β-O-diacetyl-17α-O-nicotinoyl-14-oxopremyrsinane (**1**): colorless oil; +16.4 (c 0.14 EtOAc); IR (NaCl) ν_max_ 3502, 2964, 2877, 1734, 1716, 1593, 1456, 1373, 1024, 968, 754 cm^−1^; for ^1^H and ^13^C NMR data, see Table 1; UV (EtOAc) λ_max_ (log *ε*) 260.2 (2.51); HR-ESI-MS *m/z* 736.3311 [M+Na]^+^. For additional spectra, refer to the Supplementary Materials, Figures S1–S11.
2. 3β-O-propanoyl-5α-O-benzoyl-7α,13β, 17α -O-triacetyl-14-oxopremyrsinane (**2**): colorless oil; +70.0 (c 0.03 EtOAc); IR (NaCl) ν_max_ 3498, 2954, 2929, 2856, 1739, 1456, 1261, 1038 cm^−1^; for ^1^H and ^13^C NMR data, see Table 1; UV (EtOAc) λ_max_ (log *ε*) 254.1 (2.63); HR-ESI-MS *m/z* 693.2910 [M+Na]^+^. For additional spectra, refer to the Supplementary Materials, Figures S12–S18.
3. 3β-O-propanoyl-5α-O-isobutanoyl-7α,13β, 17α -O-triacetyl-14-oxopremyrsinane (**3**): colorless oil; +10.0 (c 0.13 EtOAc); IR (KBr) ν_max_ 3492, 2974, 2941, 2879, 1734, 1716, 1456, 1371, 1342, 1232, 1192, 1142, 1038, 968, 758 cm^−1^; for ^1^H and ^13^C NMR data, see Table 1; UV (EtOAc) λ_max_ (log *ε*) 260.5 (2.57); HR-ESI-MS *m/z* 659.3050 [M+K]^+^. For additional spectra, refer to the Supplementary Materials, Figures S19–S24.
4. 3β-O-propanoyl-5α-O-isobutanoyl-7α,13β-O-diacetyl-17α-O-nicotinoyl-14-oxopremyrsinane (**4**): colorless oil; +18.3 (c 0.34 EtOAc); IR (KBr) ν_max_ 3494, 2968, 2941, 2879, 1732, 1593, 1464, 1423, 1373, 1271, 1234, 1115, 1084, 1026, 966, 756 cm^−1^; for ^1^H and ^13^C NMR data, see Table 1; UV (EtOAc) λ_max_ (log *ε*) 261.8 (2.23); HR-ESI-MS *m/z* 700.3361[M+H]^+^, 722.3162 [M+Na]^+^. For additional spectra, refer to the Supplementary Materials, Figures S25–S28.
5. 2,5,7,10,15-O-pentaacetyl-3-O-propanoyl-14-O-benzoyl-13,17-epoxy-8-myrsinene (**5**): colorless oil; for ^1^H and ^13^C NMR data, see Table 2; HR-ESI-MS *m/z* 793.20 [M+Na]^+^. For additional spectra, refer to the Supplementary Materials, Figures S29–34.

### 3.2. Structure Identification of Compounds

**Figure 2:**
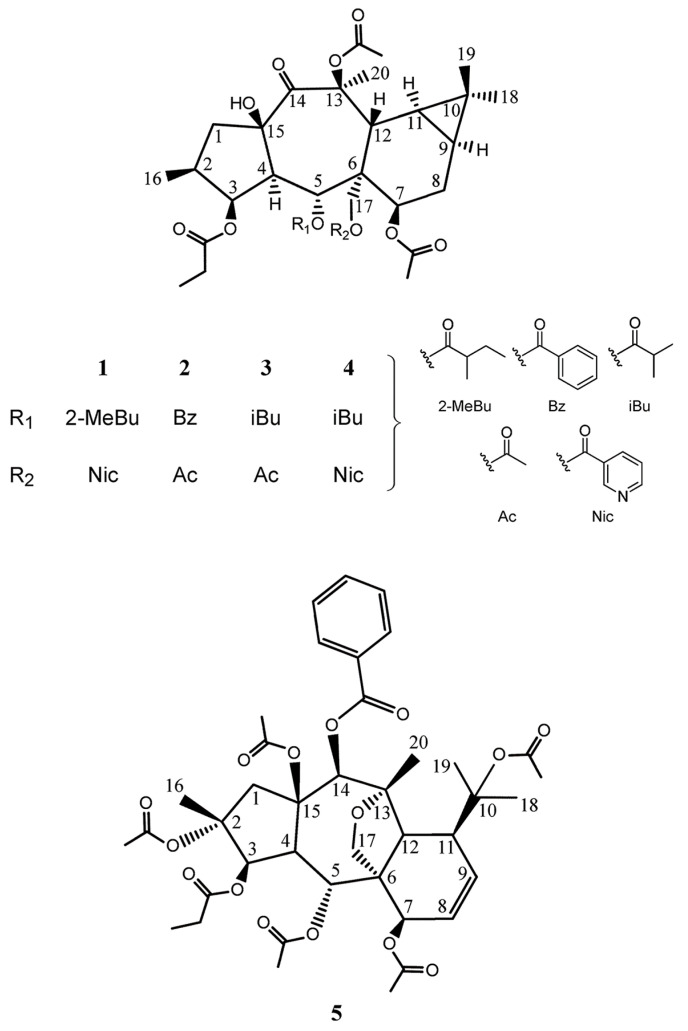
Premyrsinanes (**1**–**4**), and myrsinane diterpene (**5**) from *Euphorbia gedrosiaca*.

The chemical structure of each isolated compound had been adequately confirmed by specific spectroscopic methods such as NMR and HRMS (Figure 2).

**Figure 3:**
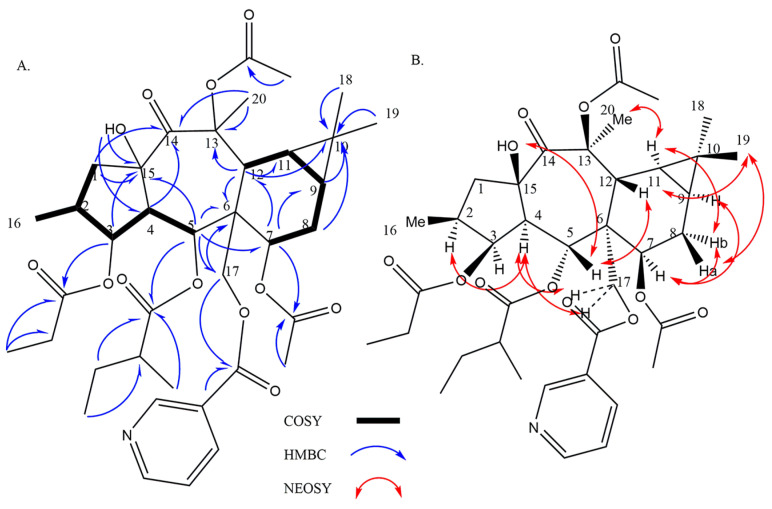
(**A**) Selected HMBC and COSY correlations and (**B**) representation of key NOESY cross peaks of **1**.

Compound **1**, isolated with optical rotation of +16.4 (c 0.14 EtOAc), was established as C_38_H_51_NO_12_ (HR-ESIMS _m/z_ 736 [M+Na]^+^; calcd 736, Δ = 3.1 ppm). Resonances at δ_C_ 165.0, 128.1, 150.7 (δ_H_: 9.16, d, *J* = 2.0 Hz), 136.9 (δ_H_: 8.19, dt, *J* = 8.0, 2.0 Hz), 123.8 (δ_H_: 7.44 (dd, *J* = 8.0, 5.0 Hz), 154.1 (δ_H_: 8.82, bd, *J* = 5.2, 1.6 Hz) belong to the nicotinoyl group at C-15 and C-24. δ_C_ 174.6, 40.8 (δ_H_: 1.95 m), 25.8 (δ_H_: 1.09–1.19/1.50–1.57, m), 11.7 (δ_H_: 0.63, t, *J* = 7.6 Hz), 14.3 (δ_H_: 0.84, d, *J* = 6.4 Hz) were indicative of 2-methylbutanoyl C-24, δ_C_ 174.2, 27.8 (δ_H_: 2.29, q, *J* = 7.6 Hz), 9.0 (δ_H_: 1.07, t, *J* = 7.2 Hz) relative to propanoyl ester, and δ_C_ 170.1, 21.4 (δ_H_: 2.11, s), as well as δ_C_ 170.8, 21.4 (δ_H_: 2.11, s) belong to two acetyl groups [14,21,22]. Without esters, the core consists of twenty carbons, including four methyls, three methylenes (one is oxygenated), eight methines (three of them attached to oxygen), and five unprotonated carbons (one ketone, and two quaternary oxycarbons). In the ^1^H-NMR, one doublet methyl (δ_H_: 0.87, d, *J* = 6.4 Hz, Me-16), three singlet methyls (δ_H_: 0.95, s, Me-19/1.06, s, Me-18/1.73, s, Me-20), two methylenes (δ_H_: 1.62, dd, *J* = 13.8, 12.0 Hz/3.17, dd, *J* = 13.8, 7.7 Hz, H-1a, b, and δ_H_: 1.86-1.94/2.18-2.24, m, H-8a, b), one oxygenated methylene (δ_H_: 4.48, d, *J* = 12.0 Hz/4.87, d, *J* = 12.0 Hz, H-17a, b), three methines including H-2 (δ_H_: 1.81-1.88, m), H-4 (δ_H_: 2.38, dd, J = 11.5, 3.6 Hz), and H-12 (δ_H_: 3.47, d, *J* = 6.7 Hz), two unusual upfield methines at δ_H_: 0.82-0.74, m/0.80-0.74, m related to H-9 and H-11 on a cyclopropane moiety, three oxymethins at δ_H_: 5.22 (dd, *J* = 3.2,3.2 Hz, H-3), 6.25 (d, *J* = 11.5 Hz, H-5), and 4.69 (bd, *J* = 6.7 Hz, H-7) were seen. ^1^H-^1^H COSY couplings determined two spin systems A (H-1—H-2(H-16) —H-3—H-4—H-5): CH_2_—CH(CH_3_)—CHO—CH—CHO and B (H-7—H-8—H-9—H-11—H-12): CHO—CH_2_—CH—CH—CH. HMBC long-range correlations of spin components at H1-5, H1-7, H1-12, and H2-17 with C-6; H1-9, H1-11, H3_-_18, H3-19 with C-10; H2_-_1/C-15, C-14; H1-12/C-13, C-14; H1-4/C-14; H3-20/C-13, C-14; H1-3/C-15 indicative of a 14-oxopremyrsinane derivative. HMBC correlations located nicotinoyl at C-17, methylbutanoyl at C-5, propanoyl at C-3, and one acetyl ester at C-7 (Figure 3A). HMBC of δ_H_: 4.44 (OH) with C-15, and C-4 located free hydroxy group at C-15, and suggested the last acetate group with no HMBC with at remaining quaternary oxycarbon C-13. For stereochemistry, taking H-4 in alpha, typical in premyrsinane diterpenes, NOESY of H-4/H-2, H-17b, as well as the small coupling constant of J_3,4_ = 3.2 Hz related to cis-relationship of H-4/H-3, determined alpha orientation of H-2, H-3, and H-17 [23,24]. A large coupling constant of J_4,5_ = 11.5 Hz indicative of trans relationship determined beta orientation of H-5 and alpha orientation of 5-O-methylbutanoyl [25,26]. NOEs of H-5/H-12, 15-OH; H-12/Me-19, and Me-19/H-8a determined the beta orientation of H-8a, H-12, 15-OH, and Me-19NOEs of H-9/H-7; H-11/H-8b, and Me-20 determined H-7, H-9, H-11, and Me-20 alpha orientation. Accordingly, compound 1 was suggested as 3β-O-propanoyl-5α-O-methylbutanoyl-7α,13β-O-diacetyl-17α-O-nicotinoyl-14-oxopremyrsinane (Figure 3B) similar to those reported by Hegazy et al. from Egyptian Plant *Euphorbia Sanctae-Catharinae* [21].

Compound **2** was isolated as a colorless oil with optical rotation +70.0 (c 0.03 EtOAc), with the molecular formula of C_36_H_46_O_12_ based on the exact ESI mass ion peak at *m/z* 693 ([M+Na]^+^, calc. 693, Δ 4.1 ppm). Resonances at δ_C_ 165.3, 130.0, 129.8 (δ_H_: 7.88, dd, *J* = 8.4, 1.5 Hz), 128.4 (δ_H_: 7.37, dd, *J* = 8.4, 7.6 Hz), 133.2 (δ_H_: 7.44, bt, *J* = 7.6 Hz), belong to the benzoyl group [14,21,27]. δ_C_ 173.7, 27.7 (δ_H_: 2.28, bq, *J* = 7.6 Hz), 8.9 (δ_H_: 1.11, t, *J* = 7.2 Hz) relative to propanoyl ester, and δ_C_ 170.3, 21.5 (δ_H_: 2.12, s), 170.8, 21.4 (δ_H_: 2.15, s), and 170.9, 21.4 (δ_H_: 1.49, s), belong to three acetate groups [14,21,22]. NMR data of polyol resembled **1** differed in 3-0-acetate instead of 3-O-nicotinoyl, and 5-O-benzoyl instead of 5-O-methylbutanoyl ester group based on HMBC correlations. In agreement with compound 4f isolated from *Euphorbia pithyusa* [28], this compound was identified as 3β-O-propanoyl-5α-O-benzoyl-7α,13β, 17α -O-triacetyl-14-oxopremyrsinane.

Compound **3** was isolated as a colorless oil with positive optical rotation +10.0 (c 0.13 EtOAc), with the molecular formula of C_33_H_48_O_12_ based on the exact ESI mass ion peak at *m/z* 659 ([M+Na]^+^, calc. 659, Δ 1.8 ppm). Resonances at δ_C_ 175.2, 34.2 (δ_H_: 2.37, m), 18.7 (δ_H_: 1.12, d, *J* = 8 Hz), 18.9 (δ_H_: 1.09 d, *J* = 8 Hz), belong to the isobutanoyl group [12,14,21,27]. δ_C_ 174.3, 27.9 (δ_H_: 2.31, q, *J* = 3.6 Hz), 9(δ_H_: 1.10, t, *J* = 6.8 Hz) relative to propanoyl ester, and δ_C_ 170.1, 21.5 (δ_H_: 2.07, s), δ_C_ 170.5, 21.4 (δ_H_: 2.10, s), and 170.8, 21.3 (δ_H_: 2.10, s), belong to three acetate groups at C-17, C-24 and C-25. NMR data of polyol resembled **2** differed in 5-O-isobutanoyl instead of the 5-O-benzoyl group [28]. Finally, compound **2** was assigned as 3β-O-propanoyl-5α-O-isobutanoyl-7α,13β, 17α -O-triacetyl-14-oxopremyrsinane.

Compound **4** as a colorless oil with positive optical rotation +18.3 (c 0.34 EtOAc), showed the molecular formula of C_37_H_49_NO_12_ based on the exact ESI mass ion peak at *m/z* 700 ([M+H]^+^, calc. 700, Δ 4.7 ppm). Resonances at δ_C_ 176.9, 34.0 (δ_H_: 2.36, m), 18.3 (δ_H_: 0.92, d, *J* = 6.8 Hz), 18.4 (δ_H_: 0.44a), belong to the isobutanoyl group [12,14,21,27]. δ_C_ 165.3, 127.5, 150.8 (δ_H_: 9.16, d, *J* = 2.0 Hz), 136.9 (δ_H_: 8.20, dt, *J* = 8.0, 1.5 Hz), 123.8 (δ_H_: 7.44 dd, *J* = 8.0, 4.8 Hz), 154.2 (δ_H_: 8.82, bd, *J* = 4.8, 1.6 Hz) belong to the nicotinoyl group [12,21]. δ_C_ 174.4, 27.8 (δ_H_: 2.27, q, *J* = 3.6 Hz), 8.9 (δ_H_: 0.57, t, *J* = 7.2 Hz) relative to propanoyl ester, and δ_C_ 170.1, 21.4 (δ_H_: 2.12, s) and 170.9, 21.4 (δ_H_: 2.12, s) belong to two acetate groups [14,21,22]. NMR data resembled **1** differed only in 5-O-isobutanoyl instead of the 5-O-methylbutanoyl group [28], which was detected as 3β-O-propanoyl-5α-O-isobutanoyl-7α,13β-O-diacetyl-17α-O-nicotinoyl-14-oxopremyrsinane. 

Compound **5**, with a molecular weight of 770 g/mol was identified as C_40_H_50_O_15_ based on HR-ESIMS m/z 793 [M+Na] + and NMR spectral data. Analyzing the ^1^HNMR, ^13^CNMR, DEPT90, and DEPT135 determined that five methyl groups at δ_H_ 1.70, 1.99, 2.05, 2.10, and 2.14 are related to five signals at δ_C_ 169.6 (7-OAc), 170.6 (10-OAc), 169.4 (2-OAc), 168.7 (5-OAc), and 170.9 (15-OAc) belonging to acetate groups. Resonances in ^1^HNMR spectra along with their relative carbon signals indicated 3-O-propanoyl ester groups at δ_H_ 2.36 (α, β), and 1.17, 14-O-benzoyl ester groups at δ_H_ 8.08 (d, *J* = 8.2 Hz), 7.45 (t, *J* = 8.2 Hz), and 7.58 (t, *J* = 8.2 Hz). Compound **5** contains 40 carbons out of which 20 of the carbon signals were associated with side chains, confirming that the remaining twenty belong to the carbons present in the core myrsinane skeleton, including four methyls, two methylenes, nine methines, and five unprotonated carbons.

HSQC and HMBC correlations confirmed the myrsinane structure of this compound in addition to seven oxygens from ketone groups attached to C-2, C-3, C-5, C-7, C-10, C-14, and C-15. The locations of ester groups were determined with HMBC, in which H-3 (δ_H:_ 5.40, d, *J* = 5.4 Hz), H-5 (δ_H:_ 5.95, dd, *J* = 11.2–1.6 Hz), H-7 (δ_H:_ 4.85, d, *J* = 6.4 Hz), and H-14 (δ_H:_ 5.82, s) were, respectively correlated with δ_C_ 173.7 (OPro), 168.69 (OAc), 169.6 (OAc), and 165.97 (OBz). These signals indicated that the propanoyl ester was located at C-3, acetyl esters at C-5 and C-7, and benzoic ester at C-14. Three signals belonging to quaternary carbons at δ_C_ 87.1 (C-2), 86.0 (C-10), and 90.0 (C-15) determined that an acetate group was situated on each of these carbons. 

Based on the data analysis and after comparison with those in the literature [29], the molecular formula of this myrsinane-type diterpene was determined to be 2,5,7,10,15-pentaacetyl-3-O-propanoyl-14-O-benzoyl-13,17-epoxy-8-myrsinene, also known as Euphorprolitherin B.

### 3.3. Determination of Cytotoxic Activity

**Figure 4:**
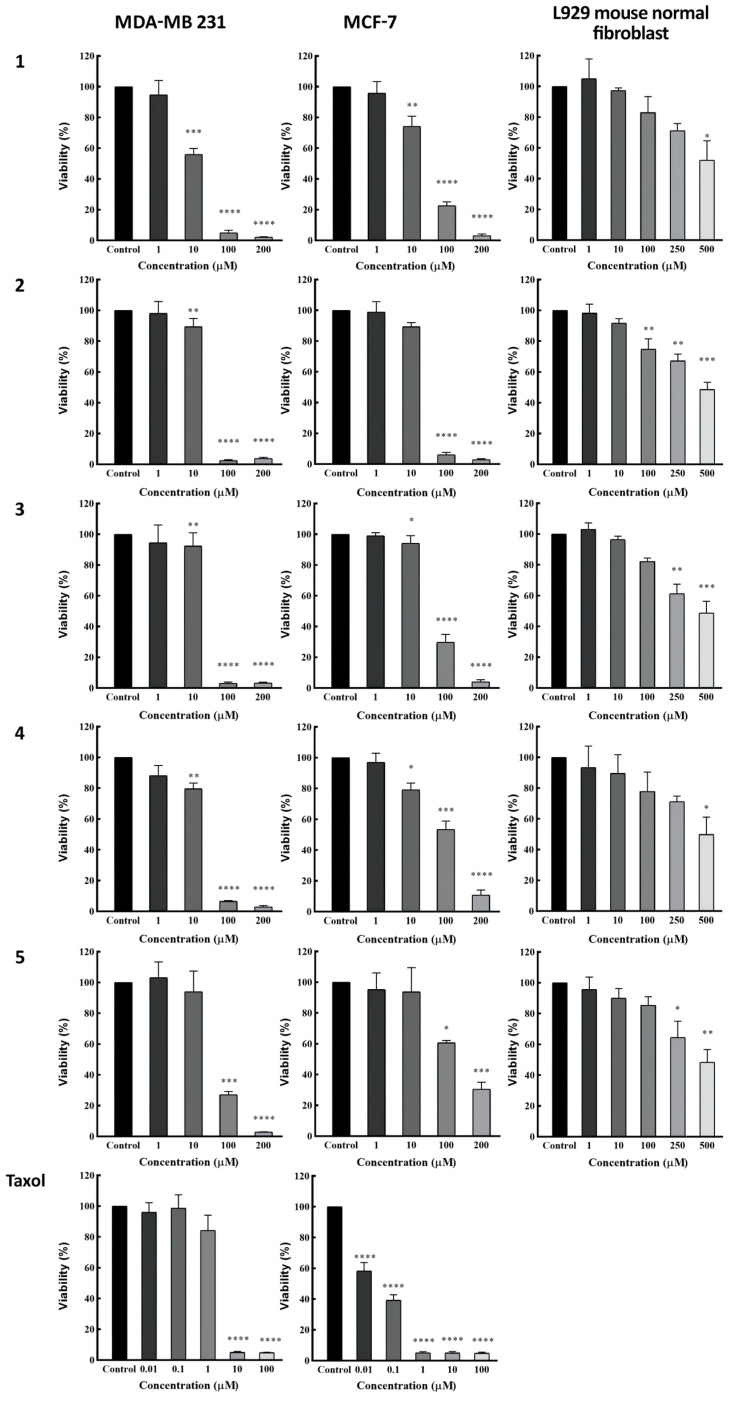
Cytotoxicity activity by using MTT viability assay against MCF-7, MDA-MB231, and L929 normal cell lines. These cells were treated with various concentrations of compounds **1–5** and Taxol as the positive control. Results are presented as the mean ± SD (n = 3). * *p* < 0.05, ** *p* < 0.01, *** *p* < 0.001, and **** *p* < 0.0001 versus control group.

Compounds **1**–**5** were tested by MTT assay to evaluate their cytotoxic activity (Table 3). All of the compounds exhibited cytotoxic effects in a dose-dependently manner with IC_50_ values of 10.8, 22.2, 24.5, 27.3, and 33.7 μM against the MDA-MB-231 (ATCCR HTB-26) breast cancer cells, and IC_50_ values of 22.2, 27.8, 62.6, 74.4, and 125.6 μM against the MCF-7 (ATCCR HTB-22) breast cancer cells, respectively (Figure 4). 

## 4. Discussion

All of the diterpenoids presented in this study are isolated and introduced for the first time from *Euphorbia gedrosiaca*, and despite their rare occurrence, compounds **1, 3,** and **4** were isolated during a study on an endemic species of Euphorbiaceae in Egypt called *Euphorbia sanctae-catharinae* and they showed moderate cytotoxicity against the proliferation of human lung (A549) and colon (Caco-2) tumor cells in different degrees [21]. Compounds **3** and **4** were also elucidated from *Euphorbia pithyusa subsp. cupanii* previously [28]. Compound **2** was elucidated from *Euphorbia dracunculoides* [9], and *Euphorbia pithyusa* [30] until now. Formerly, this compound was isolated from the roots of *Euphorbia prolifera* and showed acceptable lipid-lowering activities and TG inhibitory effects during an investigation on this plant [31]. Compound **5** has been reported in previous studies from *Euphorbia* species like *Euphorbia prolifera* [32], *Euphorbia dracunculoides* Lam [33], and *Euphorbia nematocypha* Hand.-Mazz [34].

Regarding the anti-proliferative activity of the premyrsinane diterpenes (**1–4**) in the current study, results from the MTT viability assay showed that amongst all compounds, **1** is the most effective against both cell lines, especially MDA-MB-231 with an IC_50_ value of 10.8 μM which is considerable. In general, all four diterpenes showed significant apoptotic effects against MDA-MB-231 based on the cell viability in 100 μM concentration of these bioactive agents (*p* < 0.0001). The evaluated IC_50_ from compounds **2**, **3**, and **4**, respectively, as 22.2, 24.5, and 27.3 μM, exhibits that they are similarly effective against MDA-MB-231. Compound **2** is the second most effective one based on our findings, which had quite the same efficacy on both cell lines. Although the effectiveness of **1** against MCF-7 did not differ significantly from **2**, both were reasonably potent in 100 μM concentration with P values less than 0.0001. Compounds **3** and **4** demonstrated nearly similar MTT test results with almost three times higher cytotoxicity against the MDA-MB-231 cell line compared to MCF-7, which can be considered a moderate activity and not as potent as other responses in this study. This comparison shows that this similarity is due to their chemical structural analogy, which only differs at C-17 by replacing an acetyl ester group with a nicotinoyl group. Compound **5** was also tested for its cytotoxicity against these two cell lines but showed less potency than the other four compounds. However, the efficacy is acceptable against the MDA-MB-231 with an IC_50_ value of 33.7 μM, and can be worthy of consideration, but the IC_50_ value of 125.6 μM against MCF-7 shows low activity. The comparison of MTT assay data also reveals that the myrsinane diterpenes have less efficacy as anti-proliferative agents against these two cancer cell lines. New findings about the structure–activity relationship for cytotoxic efficacy can be helpful to approach the best chemical structure in therapeutic applications.

All of the isolated compounds were also tested on mouse normal fibroblast cell lines to prove their safety, and the results were promising, while none of them demonstrated toxicity at concentrations up to 250 µM towards L929 normal cell line.

Former studies concerning the evaluation of cytotoxicity and possible therapeutic application of diterpenoids in breast cancer treatment have also revealed valuable results as well. In an identical study on *Euphorbia aleppica*, two new cytotoxic premyrsinane diterpenes were introduced and assessed for their anti-cancer effects on MCF-7 and MDA-MB-231 cell lines which determined their probable potency [16]. Similarly, another investigation on cyclomyrsinane diterpenes from *Euphorbia* species proved the potential activity of compounds that had structural similarities to the ones elucidated in the present research against the same breast tumor cell lines [35].

## 5. Conclusions

Five diterpenoids were determined from *E.gedrosiaca* and evaluated for their anti-proliferative properties against MCF-7 and MDA-MB231 cell lines. The MTT viability assay proves that compound **1** is the most effective against both cell lines, especially MDA-MB-231. We can conclude that these diterpenes show better apoptotic effects against MDA-MB-231 than MCF-7, and it is clear that the premyrsinane skeletal structure improves the cytotoxic activity of compounds against breast cancer cell lines. On the other hand, the results displayed the safety of the isolated compounds against normal cells, which can be beneficial in drug development.

Considering all of the above mentioned, in order to achieve valuable and novel discoveries as practical anti-breast cancer treatments, it is worthwhile to survey and precisely determine the most effective bioactive chemical structure of this type of diterpenoids and detect the most potent apoptotic agent by comparing the replacement of different chemical groups and assess their effects.

## Figures and Tables

**Table 1:** ^1^H (400 MHz) and ^13^C (100 MHz) NMR data of compounds **1–4** in CDCl_3_.

Position	1		2		3		4	
	*δ*_H_ (mult., *J* in Hz)	*δ*_C_ (Type)	*δ*_H_ (mult., *J* in Hz)	*δ*_C_ (Type)	*δ*_H_ (mult., *J* in Hz)	*δ*_C_ (Type)	*δ*_H_ (mult., *J* in Hz)	*δ*_C_ (Type)
1a	1.62 (dd, 13.8, 12.0)	42.9 (CH_2_)	1.65 (a ^1^)	43.0 (CH_2_)	1.62 (a)	42.9 (CH_2_)	1.63 (a)	43.0 (CH_2_)
1b	3.17 (dd, 13.8, 7.7)	-	3.16 (dd, 13.6, 7.6)	-	3.15 (dd, 13.6, 7.6)	-	3.18 (dd, 13.6, 7.6)	-
2	1.81–1.88 (m)	37.5 (CH)	1.78–1.82 (m)	37.4 (CH)	1.72–1.79 (m)	37.5 (CH)	1.81–1.88 (m)	37.5 (CH)
3	5.22 (t, 3.2)	78.4 (CH)	5.38 (t, 3.6)	78.3 (CH)	5.27 (t, 3.2)	78.4 (CH)	5.24 (t, 3.2)	78.4 (CH)
4	2.38 (dd, 11.5, 3.6)	50.6 (CH)	2.39 (dd, 11.6, 3.6)	50.4 (CH)	2.40 (dd, 11.6, 3.2)	50.4 (CH)	2.32 (dd, 11.6, 3.2)	50.6 (CH)
5	6.25 (d, 11.5)	69.1 (CH)	6.38 (d, 11.6)	70.0 (CH)	6.19 (d, 11.6)	68.9 (CH)	6.23 (d, 11.6)	69.1 (CH)
6	-	47.7 (C)	-	47.9 (C)	-	47.5 (C)	-	47.8 (C)
7	4.69 (bd, 6.7)	70.8 (CH)	4.80 (d, 6.8)	70.8 (CH)	4.51 (d, 6.8)	70.7 (CH)	4.71 (d, 6.8)	70.8 (CH)
8a	1.86–1.94 (m)	22.5 (CH_2_)	1.83–1.90 (m)	22.2 (CH_2_)	1.78–1.86 (m)	22.2 (CH_2_)	1.88–1.96 (m)	22.5 (CH_2_)
8b	2.18–2.24 (m)	-	2.19–2.25 (m)	-	2.18–2.24 (m)	-	2.16–2.22 (m)	-
9	0.74–0.82 (m)	19.0 (CH)	0.69–0.77 (m)	19.1 (CH)	0.71–0.80 (m)	18.90 (CH)	0.75-.083 (m)	19.1 (CH)
10	-	18.4 (C)	-	18.4 (C)	-	18.2 (C)	-	18.56 (C)
11	0.74–0.80 (m)	23.9 (CH)	0.69–0.77 (m)	24.0 (CH)	0.71–0.80 (m)	23.9 (CH)	0.75–0.83 (m)	24.0 (CH)
12	3.47 (d, 6.7)	35.0 (CH)	3.52 (d, 6.4)	35.2 (CH)	3.39 (d, 6.4)	34.9 (CH)	3.49 (d, 6.8)	35.1 (CH)
13	-	85.8 (C)	-	85.9 (C)	-	86.0 (C)	-	85.9 (C)
14	-	204.4 (C)	-	204.4 (C)	-	204.5 (C)	-	204.8 (C)
15	-	84.2 (C)	-	84.3 (C)	-	84.1 (C)	-	84.2 (C)
16	0.87 (d, 6.4)	14.7 (CH_3_)	0.86 (d, 6.4)	14.0 (CH_3_)	0.88 (d, 6.4)	14.2 (CH_3_)	0.88 (d, 7.2)	14.2 (CH_3_)
17a	4.48 (d, 12.0)	64.6 (CH_2_)	4.31 (d, 11.6)	62.9 (CH_2_)	4.35 (d, 11.6)	63.6 (CH_2_)	4.51 (d, 11.6)	64.5(CH_2_)
17b	4.87 (d, 12.0)	-	4.69 (d, 11.6)	-	4.42 (d, 11.6)	-	4.88 (d, 12.0)	-
18	1.06 (s)	29.6 (CH_3_)	1.06 (s)	29.6 (CH_3_)	1.05 (s)	29.6 (CH_3_)	1.07 (s)	29.6 (CH_3_)
19	0.95 (s)	14.9 (CH_3_)	0.95 (s)	15.0 (CH_3_)	0.91 (s)	14.9 (CH_3_)	0.95 (s)	15.0 (CH_3_)
3-OPro	-	174.2	-	173.7	-	174.3	-	174.4
	2.34 (q, 7.6)	27.8	2.30 (q, 7.2)	27.7	2.31 (q, 7.2)	27.9	2.32 (q, 7.6)	27.8
	0.63 (t, 7.6)	9.0	0.98 (t, 7.2)	8.9	0.74-0.79 (m)	9.0	0.57 (t, 7.2)	8.9
5-OMeBu	-	174.6	-	-	-	-	-	-
	1.95 (m)	40.8	-	-	-	-	-	-
	1.09–1.19 (m)	25.8	-	-	-	-	-	-
	1.50–1.57 (m)							
	0.63 (t, 7.6)	11.7	-	-	-	-	-	-
	0.84 (d, 6.4)	14.3	-	-	-	-	-	-
5-OiBut	-	-	-	-	-	175.2	-	176.9
	-	-	-	-	2.38 (m)	34.2	2.36 (m)	34.0
	-	-	-	-	1.12 (d, 7.8)	18.7	0.92 (d, 6.8)	18.3
	-	-	-	-	1.09 (d, 7.8)	18.9	0.44 (a)	18.4
5-OBz	-	-	-	129.8	-	-	-	-
	-	-	7.87 (bd, 7.8)	128.4	-	-	-	-
	-	-	7.37 (t, 7.8)	133.2	-	-	-	-
	-		7.51 (t, 7.6)		-		-	
7-OAc	-	170.1	-	170.8	-	170.25	-	170.1
	2.11 (s)	21.4	2.15 (s)	21.4	2.10 (s)	21.4	2.12 (s)	21.4
13-OAc	-	170.8	-	170.9	-	170.8	-	170.9
	2.11 (s)	21.4	1.49 (s)	20.6	2.10 (s)	21.3	2.12 (s)	21.4
17-OAc	-	-		170.3		170.1		-
	-	-	2.12 (s)	21.5	2.07 (s)	21.5	-	-
17-ONic	-	165.0	-	-.	-	-	-	165.3
	9.16 (bs)	128.1	-	-	-	-	9.16 (d, 2.0)	127.5
	-	150.7	-	-	-	-	-	150.8
	8.19 (bd, 8.0)	136.9	-	-	-	-	8.20 (dt, 8.0, 1.5)	136.9
	7.44 (dd, 8.0, 5.0)	123.8	-	-	-	-	7.44 (dd, 8.0, 4.8)	123.8
	8.82 (bd, 5.2)	154.1	-	-	-	-	8.82 (bd, 4.8, 1.6)	154.2

^1^ a: overlap.

**Table 2:** ^1^H (400 MHz) and ^13^C (100 MHz) NMR data of compound **5** in CDCl_3_.

Position	*δ*_H_ (mult., *J* in Hz)	*δ*_C_ (Type)	Position	*δ*_H_ (mult., *J* in Hz)	*δ*_C_ (Type)
1a	3.31 (d, 17.2)	47.2 (CH_2_)	20	1.23 (s)	24.5 (CH_3_)
1b	2.36 (d, 17.2)	-	2-OAc	-	169.4
2	-	87.1 (C)		2.05 (s)	22.7
3	5.40 (bd, 5.4)	78.3 (CH)	3-OPro	-	173.7
4	3.74 (dd, 11.2, 4)	47.6 (CH)		2.36 (m)	28.2
5	5.95 (dd, 11.2, 1.6)	68.7 (CH)		1.17 (s)	8.9
6	-	53.7 (C)	5-OAc	-	168.7
7	4.85 (d, 6.4)	63.1 (CH)		2.10 (s)	21.4
8	6.19 (dd, 9.9, 6.6)	126.0 (CH)	7-OAc	-	169.6
9	6.23 (bd, 10.4)	131.8 (CH)		1.70 (s)	22.4
10	-	86.0 (C)	10-OAc	-	170.6
11	3.18 (bd, 5)	44.8 (CH)		1.99 (s)	21.0
12	3.20 ^a^	37.2 (CH)	14-OAc	-	166.0
13	-	90.2 (C)		-	130.1
14	5.82 (s)	73.3 (CH)		8.08 (d, 8.2)	130.3
15	-	90.0 (C)		7.45 (t, 8.2)	128.6
16	1.31 (s)	18.9 (CH_3_)		7.58 (t, 8.2)	133.5
17a	4.16 (d, 8.8)	70.0 (CH_2_)	15-OAc	-	170.9
17b	3.52 (dd, 8.6, 1.6)	-		2.14 (s)	22.5
18	1.64 (s)	25.4 (CH_3_)			
19	1.54 (s)	21.1 (CH_3_)			

a: overlap.

**Table 3:** Cytotoxic activity [IC_50_ (μM)] of compounds **1–5**.

Tested Cell Line	1	2	3	4	5
MDA-MB-231	10.8 ± 2.1	22.2 ± 4.0	24.5 ± 3.8	27.3 ± 3.2	33.7 ± 4.6
MCF-7	22.2 ± 2.4	27.8 ± 2.5	62.6 ± 4.9	74.4 ± 5.2	125.6 ± 4.2
L929 (normal)	528.3 ± 23.8	553.1 ± 26.7	433.1 ± 22.1	716.4 ± 26.9	596.3 ± 23.5

Tested cell lines were MDA-MB-231 (ATCCR HTB-26), and MCF-7 (ATCCR HTB-22) breast cancer cells. L929 mouse fibroblast cell line was used as a normal cell line. Results are presented as the mean ± SD (n = 3).

## Data Availability

The data presented in this study are available in the article.

## Supplementary Materials

The following supporting information can be downloaded at: https://www.mdpi.com/article/10.3390/metabo13020225/s1, Figure S1. 1H NMR spectrum of (**1**), Figure S2. 13C NMR spectrum of (**1**), Figure S3. 13C DEPT135 spectrum of (**1**), Figure S4. 13C DEPT90 spectrum of (**1**), Figure S5. HSQCGP spectrum of (**1**), Figure S6. HMBCGP spectrum of (**1**), Figure S7. DQF-COSY spectrum of (**1**), Figure S8. FT-IR spectrum of (**1**), Figure S9. (A) Selected HMBC and COSY correlations and (B) representation of key NOESY cross peaks of (**1**), Figure S10. NOESY spectrum of (**1**), Figure S11. HR-ESI-MS spectrum of (**1**), Figure S12. 1H NMR spectrum of (**2**), Figure S13. 13C NMR spectrum of (**2**), Figure S14. 13C DEPT135 spectrum of (**2**), Figure S15. 13C DEPT90 spectrum of (**2**), Figure S16. HMBCGP spectrum of (**2**), Figure S17. HR-ESI-MS spectrum of (**2**), Figure S18. FT-IR spectrum of (**2**), Figure S19. 1H NMR spectrum of (**3**), Figure S20. 13C NMR spectrum of (**3**), Figure S21. 13C DEPT135 spectrum of (**3**), Figure S22. 13C DEPT90 spectrum of (**3**), Figure S23. HR-ESI-MS spectrum of (**3**), Figure S24. FT-IR spectrum of (**3**), Figure S25. 1H NMR spectrum (**4**), Figure S26. 13C NMR spectrum of (**4**), Figure S27. HR-ESI-MS spectrum of (**4**), Figure S28. FT-IR spectrum of (**4**), Figure S29. 1H NMR spectrum of (**5**), Figure S30. 13C NMR spectrum of (**5**), Figure S31. 13C DEPT135 spectrum of (**5**), Figure S32. 13C DEPT90 spectrum of (**5**), Figure S33. HSQCGP spectrum of (**5**), Figure S34. HMBCGP spectrum of (**5**), Figure S35. HPLC chromatogram of fraction 5 corresponding to (**1**), (**2**) and (**3**), Figure S36. HPLC chromatogram of fraction 10-1 corresponding to (**4**), Figure S37. HPLC chromatogram of fraction 9 corresponding to (**5**).

## Abbreviations

**Term**	**Definition**
COSY	Correlated Spectroscopy
DMSO	Dimethyl sulfoxide
ELISA	Enzyme-linked Immunosorbent Assay
EtOAc	Ethyl acetate
HMBC	Heteronuclear Multiple Quantum Correlation
HPLC	High Performance Liquid Chromatography
HRMS	High-resolution Mass Spectrometry
HSQC	Heteronuclear Single Quantum Correlation
MPLC	Medium Pressure Liquid Chromatography
MTT	Microculture Tetrazolium Test
NMR	Nuclear Magnetic Resonance
NOESY	Nuclear Overhauser Enhancement Spectroscopy
TLC	Thin Layer Chromatography
