# Supplementary material for: Diterpenoids from Euphorbia gedrosiaca as Potential Anti-Proliferative Agents against Breast Cancer Cells
Source: Metabolites. 2023 Feb 3;13(2):225. doi: 10.3390/metabo13020225 (PMC9964718; doi:10.3390/metabo13020225)
Supplement: Supplementary file 1 [file metabolites-13-00225-s001.zip › metabolites-2152454-Supplementary.pdf]

Supplementary materials

List of Contents

Figure S1. ^{1}H **NMR (400 MHz) spectrum of compound 1**

Figure S2. ^{13}C **NMR (100 MHz) spectrum of compound 1**

Figure S3. ^{13}C **DEPT135 (100 MHz) spectrum of compound 1**

Figure S4. ^{13}C **DEPT90 (100 MHz) spectrum of compound 1**

Figure S5. HSQCGP (400 MHz) spectrum of compound 1

Figure S6. HMBCGP (400 MHz) spectrum of compound 1

Figure S7. DQF-COSY (400 MHz) spectrum of compound 1

Figure S8. FT-IR spectrum of compound 1

Figure S9. (A) Selected HMBC and COSY correlations and (B) representation of key NOESY cross peaks of compound 1

Figure S10. NOESY (400 MHz) spectrum of compound 1

Figure S11. HR-ESI-MS spectrum of compound 1

Figure S12. ^{1}H **NMR (400 MHz) spectrum of compound 2**

Figure S13. ^{13}C **NMR (100 MHz) spectrum of compound 2**

Figure S14. ^{13}C **DEPT135 (100 MHz) spectrum of compound 2**

Figure S15. ^{13}C **DEPT90 (100 MHz) spectrum of compound 2**

Figure S16. HMBCGP (400 MHz) spectrum of compound 2

Figure S17. HR-ESI-MS spectrum of compound 2

Figure S18. FT-IR spectrum of compound 2

Figure S19. ^{1}H **NMR (400 MHz) spectrum of compound 3**

Figure S20. ^{13}C **NMR (100 MHz) spectrum of compound 3**

Figure S21. ^{13}C **DEPT135 (100 MHz) spectrum of compound 3**

Figure S22. ^{13}C **DEPT90 (100 MHz) spectrum of compound 3**

Figure S23. HR-ESI-MS spectrum of compound 3

Figure S24. FT-IR spectrum of compound 3

Figure S25. ^{1}H **NMR (400 MHz) spectrum compound 4**

Figure S26. ^{13}C **NMR (100 MHz) spectrum of compound 4**

Figure S27. HR-ESI-MS spectrum of compound 4

Figure S28. FT-IR spectrum of compound 4

Figure S29. ^{1}H NMR (400 MHz) spectrum of compound 5

Figure S30. ^{13}C NMR (100 MHz) spectrum of compound 5

Figure S31. ^{13}C DEPT135 (100 MHz) spectrum of compound 5

Figure S32. ^{13}C DEPT90 (100 MHz) spectrum of compound 5

Figure S33. HSQCGP (400 MHz) spectrum of compound 5

Figure S34. HMBCGP (400 MHz) spectrum of compound 5

Figure S35. HPLC chromatogram of fraction 5 showing peaks corresponding to compounds 1, 2 and 3

Figure S36. HPLC chromatogram of fraction 10-1 showing peak corresponding to compound 4

Figure S37. HPLC chromatogram of fraction 9 showing peak corresponding to compound 5

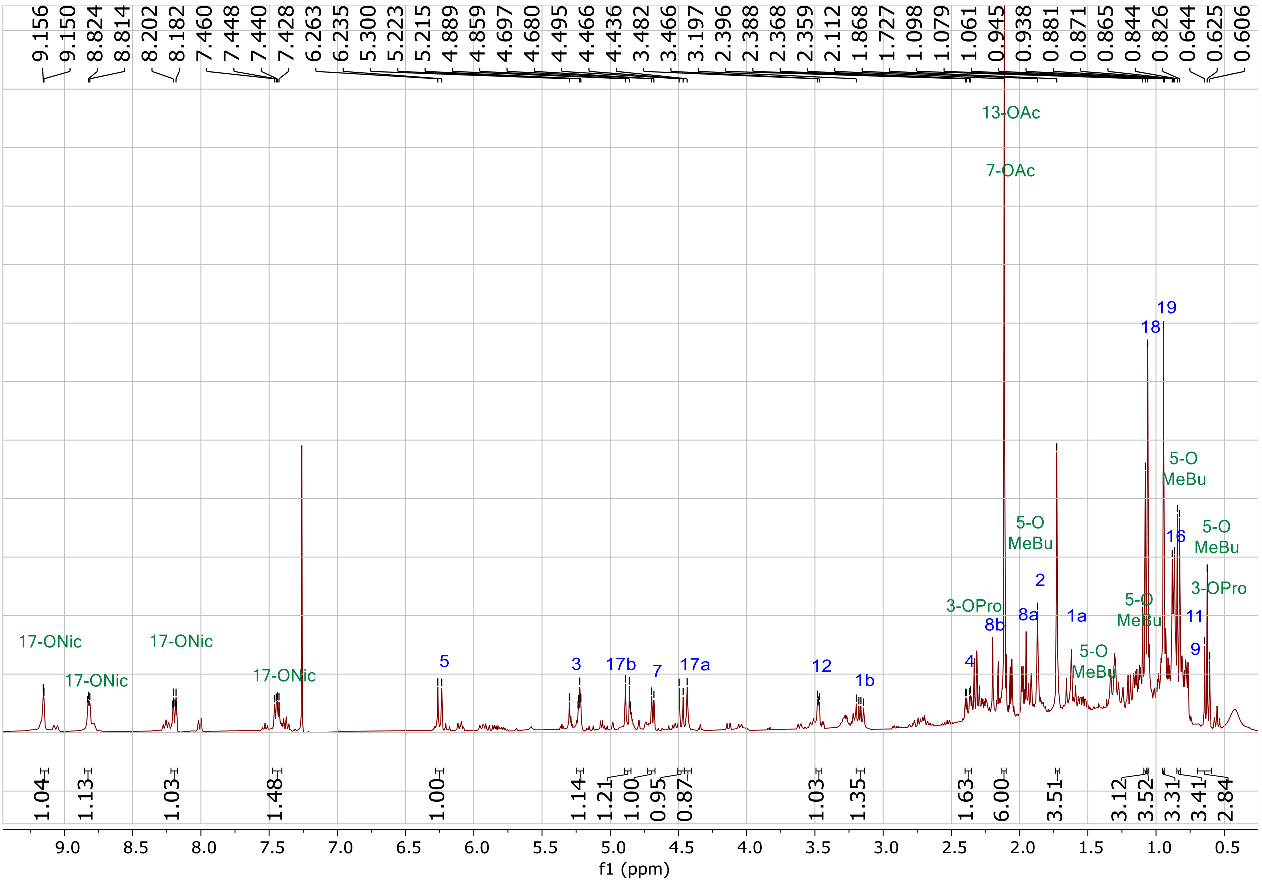

Figure S1. ^{1}H NMR (400 MHz) spectrum of compound **1**

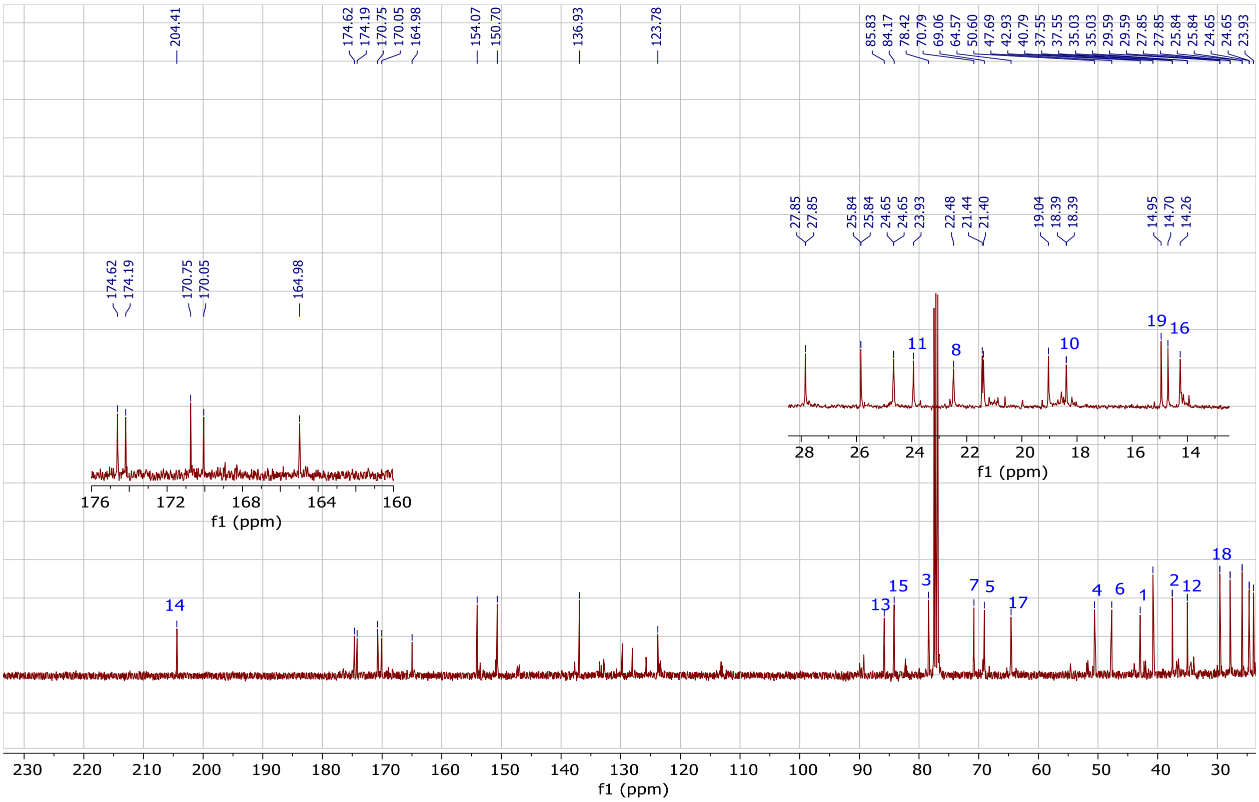

Figure S2. ^{13}C NMR (100 MHz) spectrum of compound **1**

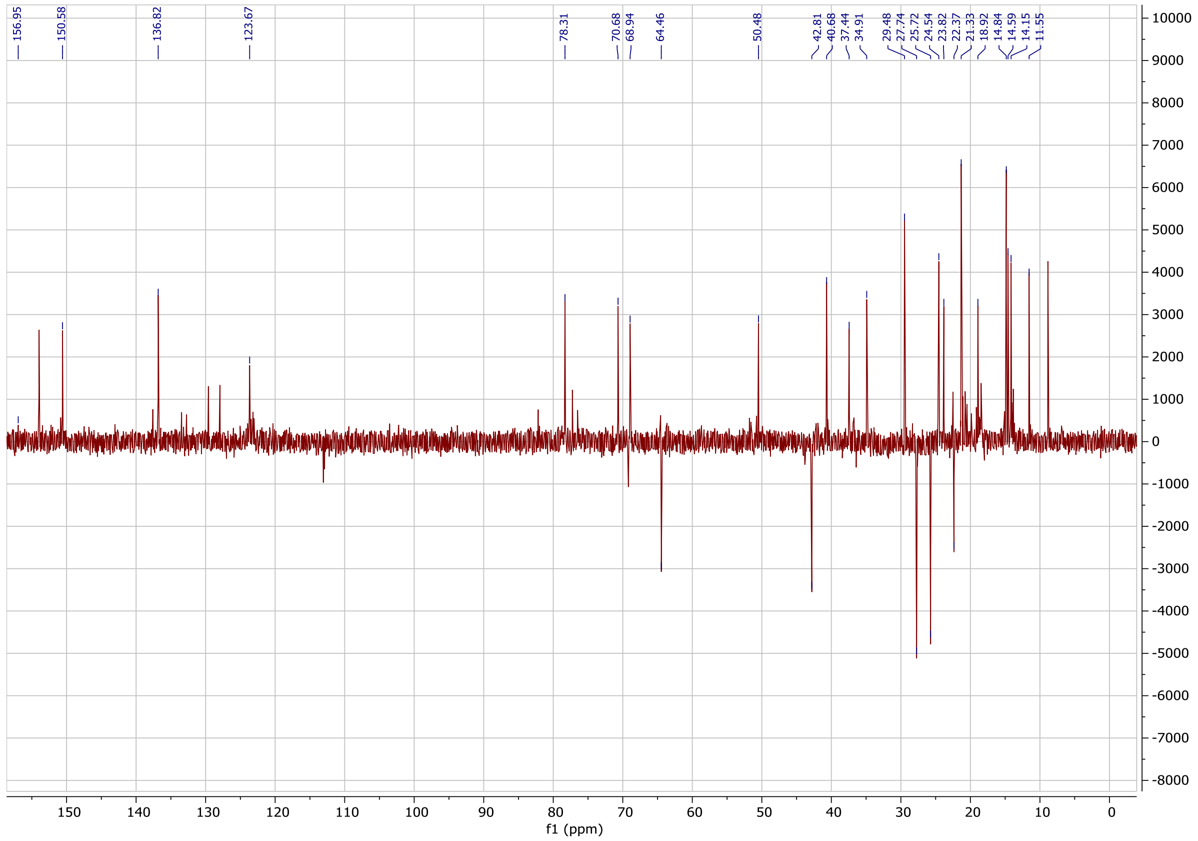

Figure S3. ^{13}C DEPT135 (100 MHz) spectrum of compound **1**

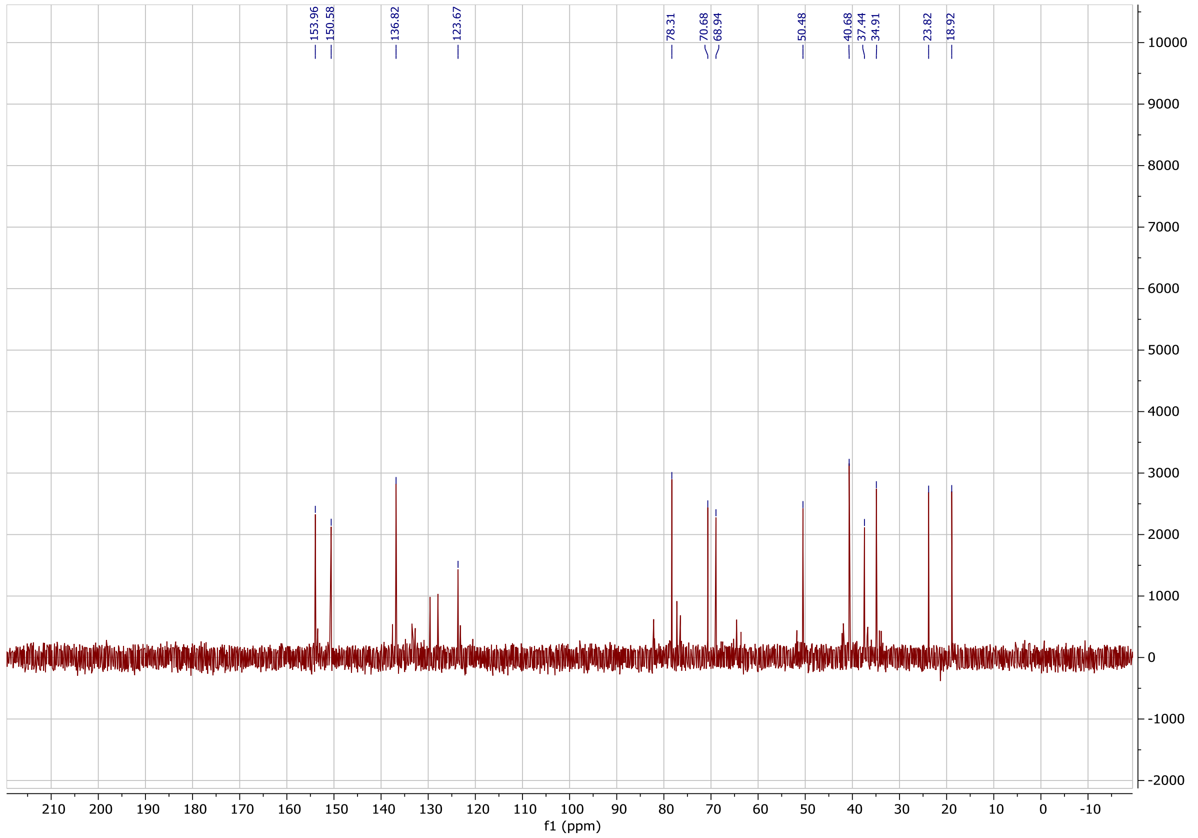

Figure S4. ^{13}C DEPT90 (100 MHz) spectrum of compound **1**

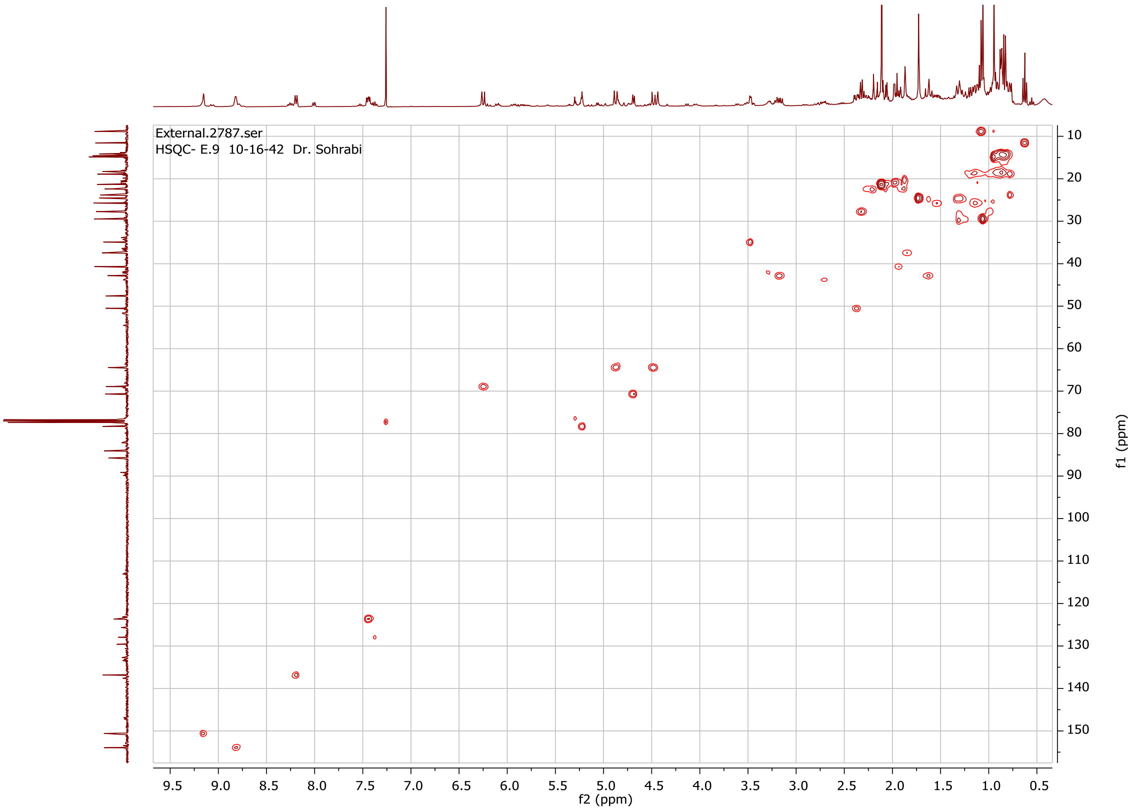

Figure S5. HSQCGP (400 MHz) spectrum of compound **1**

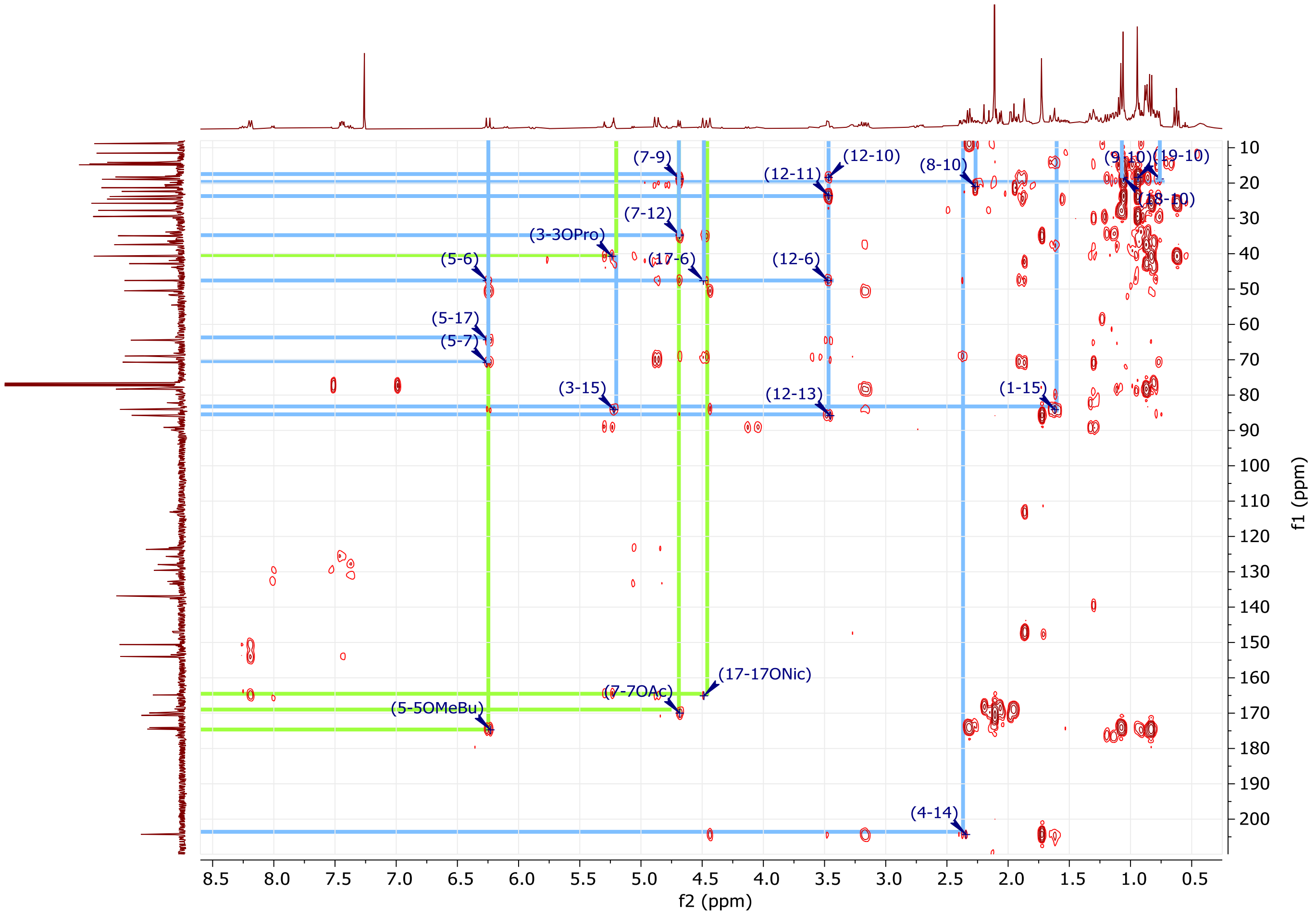

Figure S6. HMBCGP (400 MHz) spectrum of compound **1**

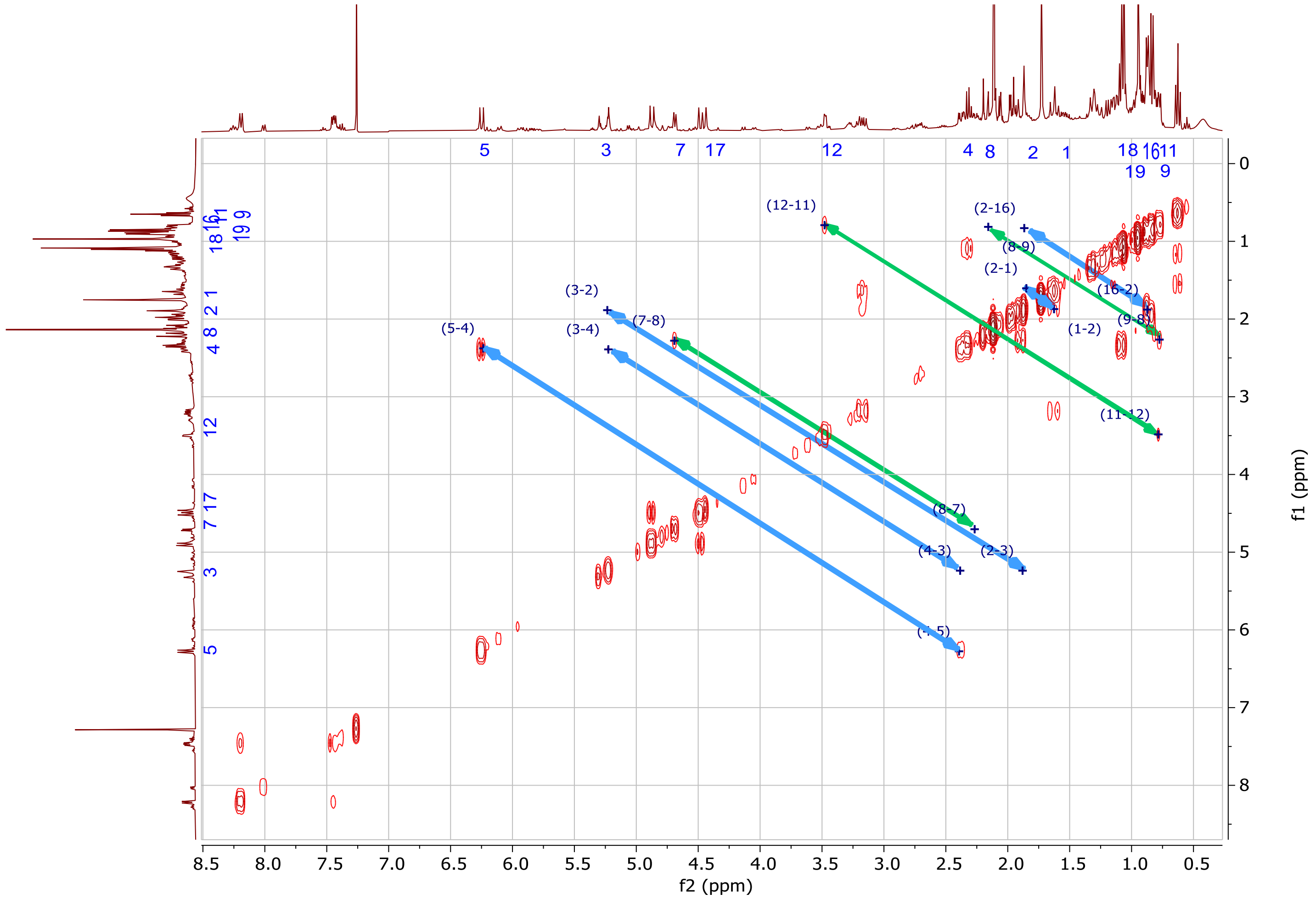

Figure S7. DQF-COSY (400 MHz) spectrum of compound **1**

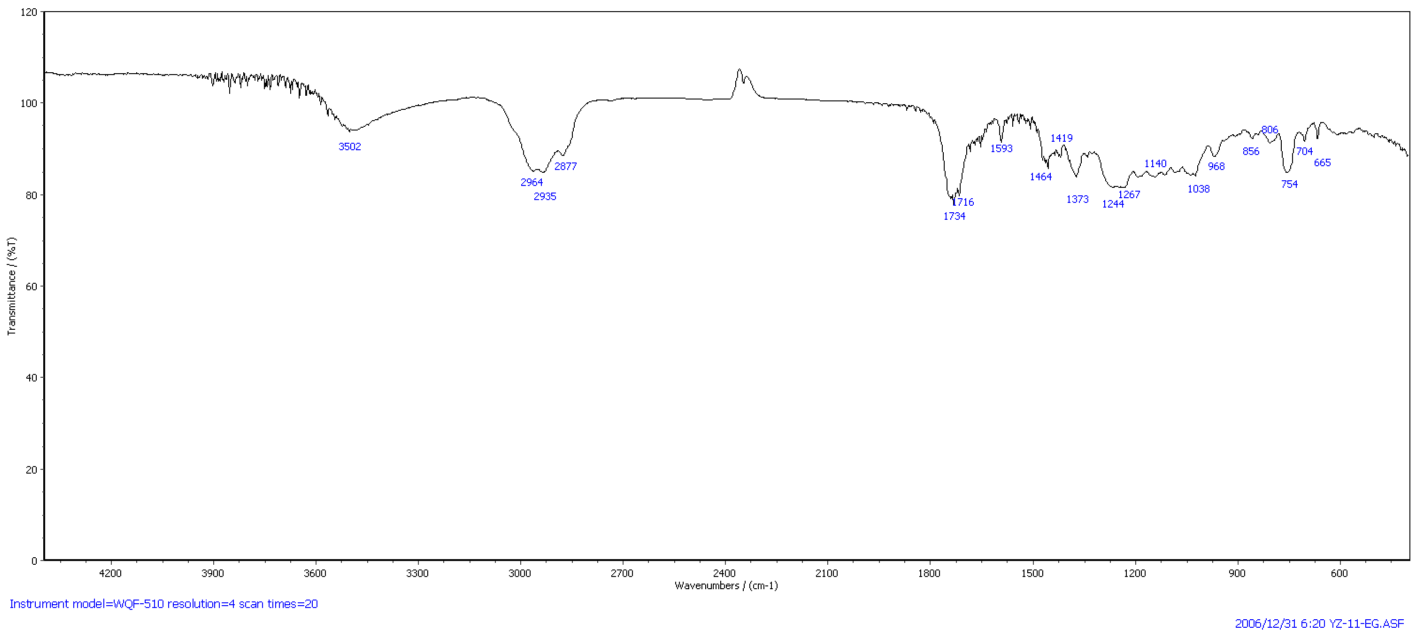

Figure S8. FT-IR spectrum of compound **1**

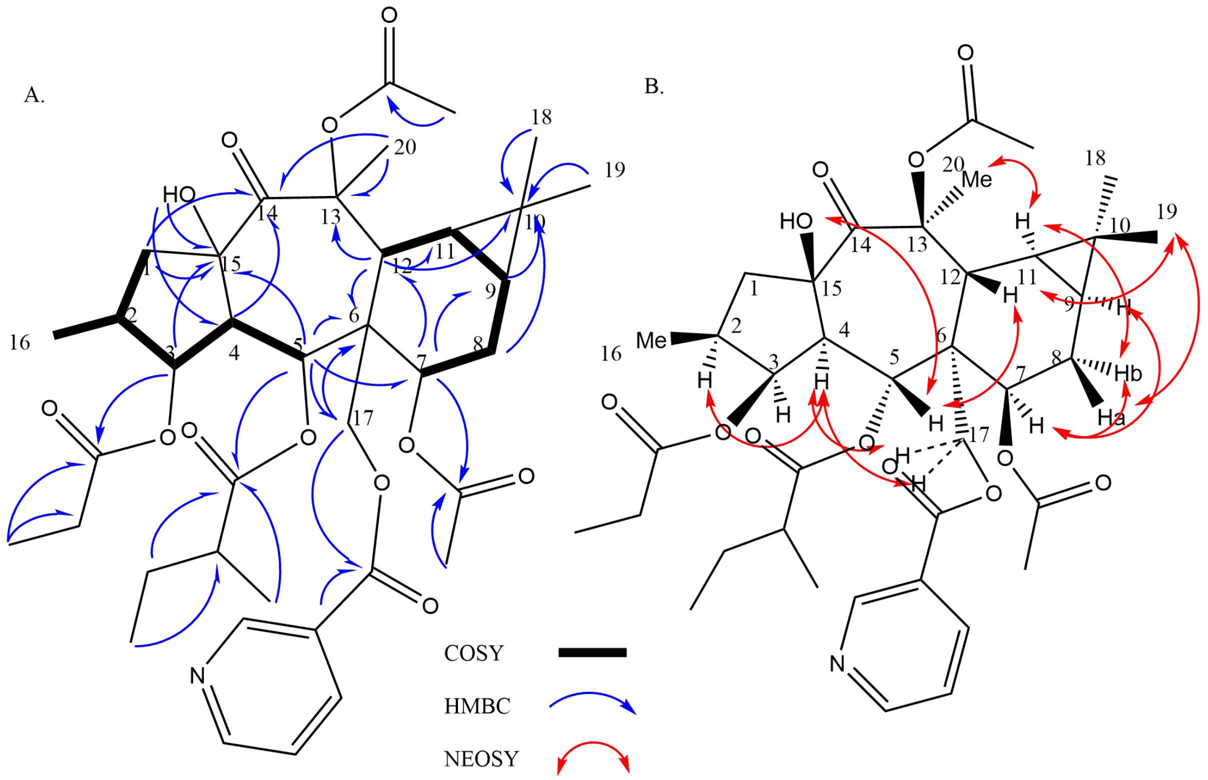

Figure S9.(A) Selected HMBC and COSY correlations and (B) representation of key NOESY cross peaks of compound **1**

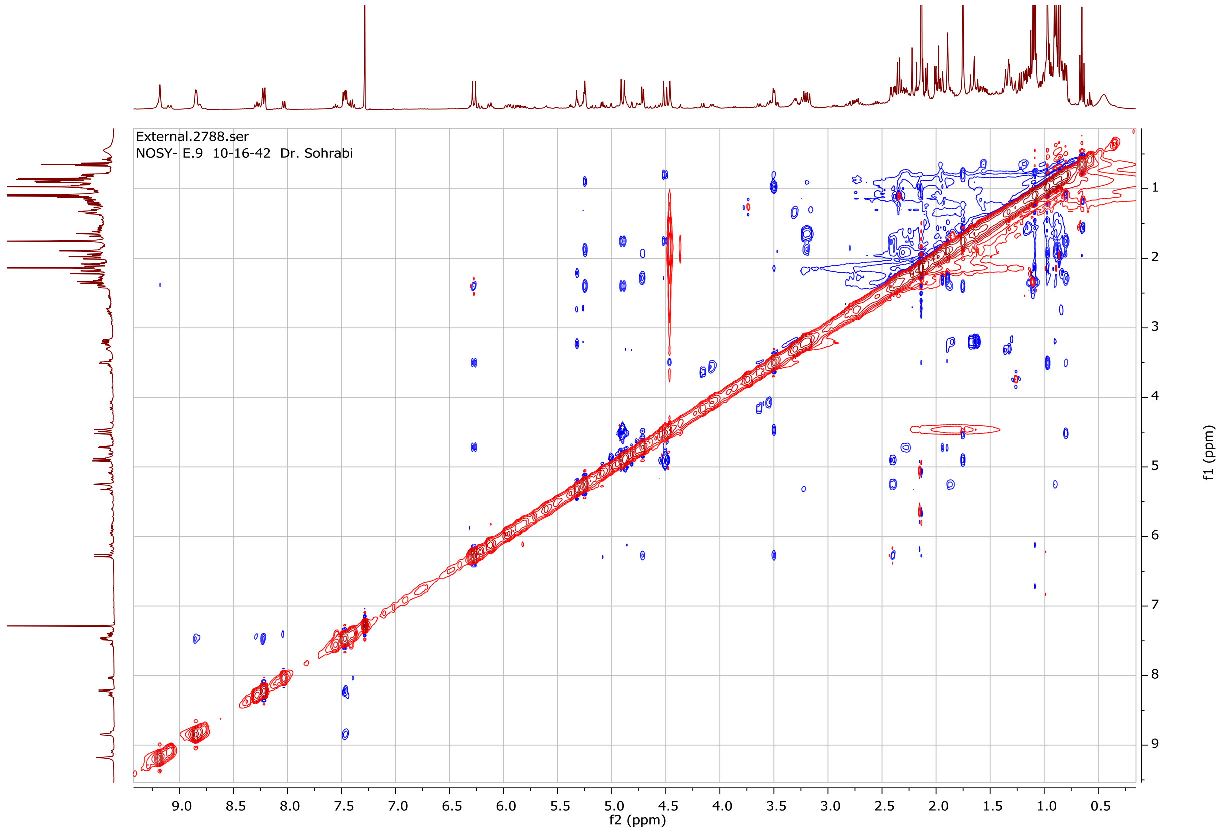

Figure S10. NOESY (400 MHz) spectrum of compound **1**

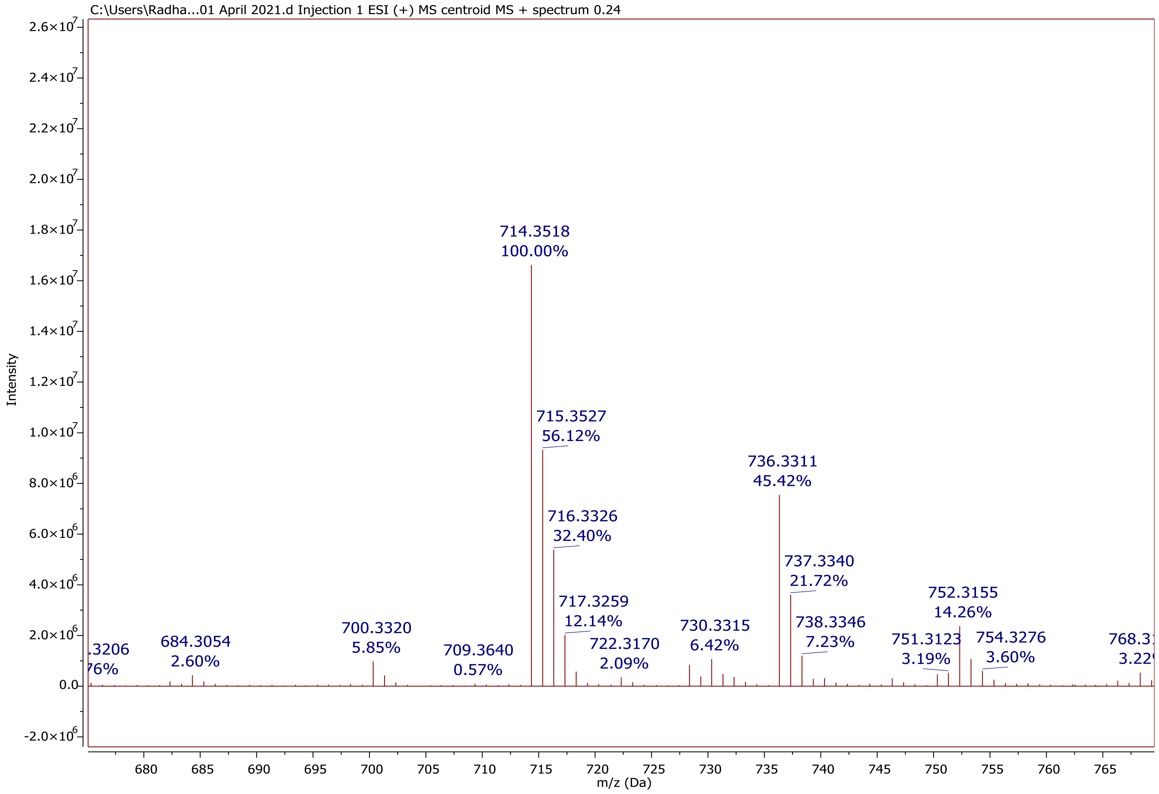

Figure S11. HR-ESI-MS spectrum of compound **1**

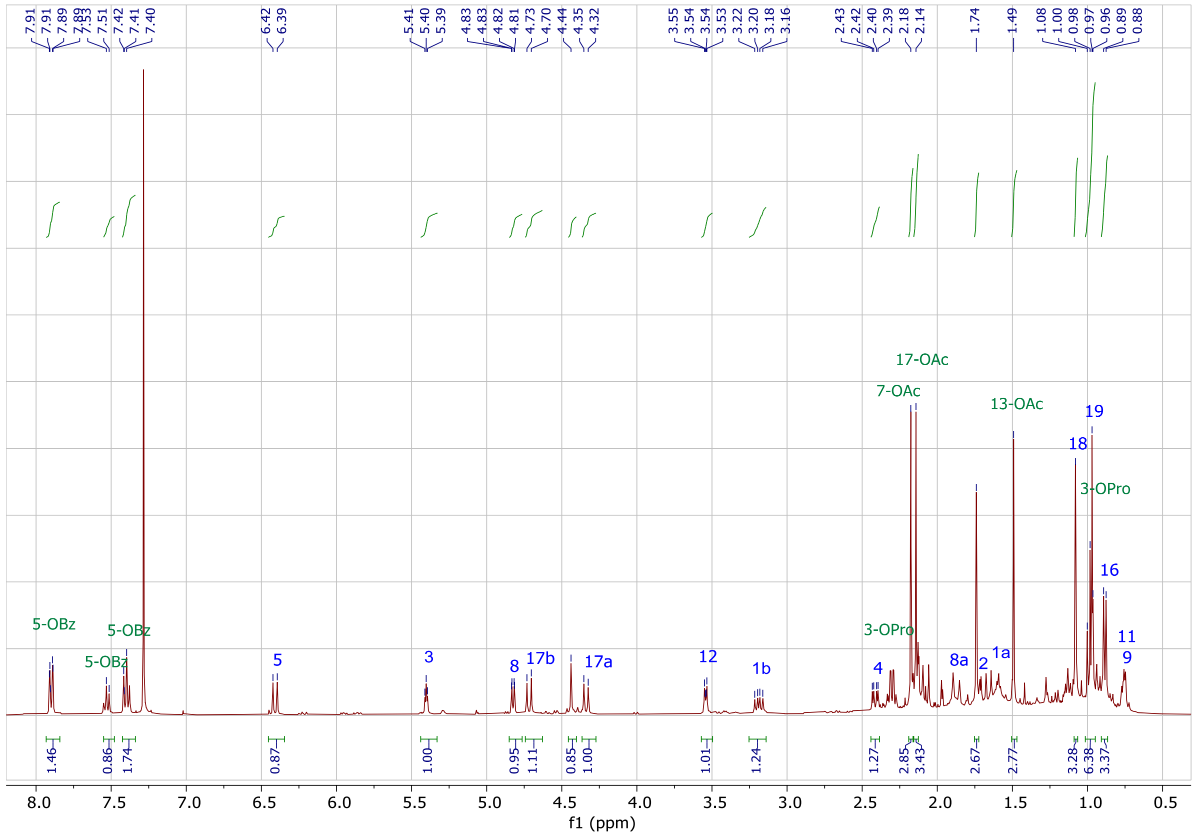

Figure S12. 1H NMR (400 MHz) spectrum of compound **2**

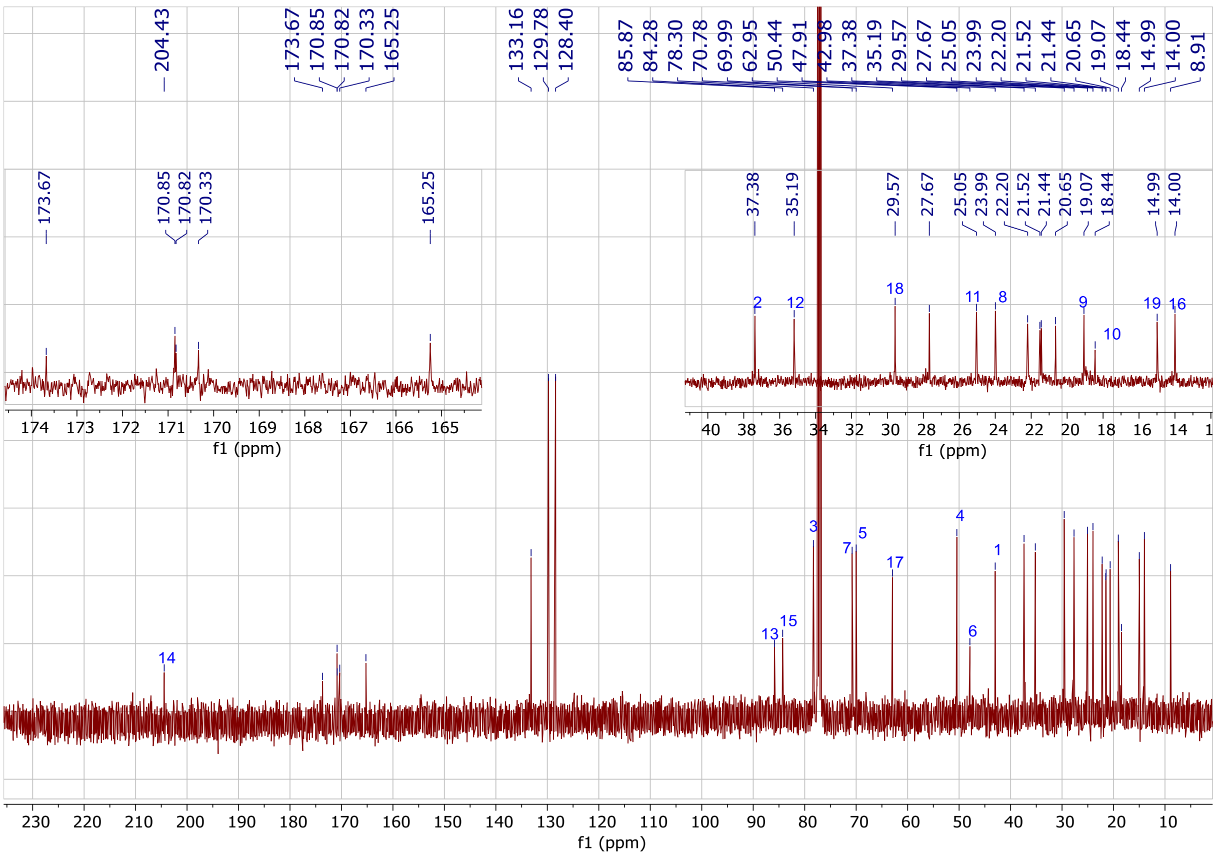

Figure S13. ^{13}C NMR (100 MHz) spectrum of compound 2

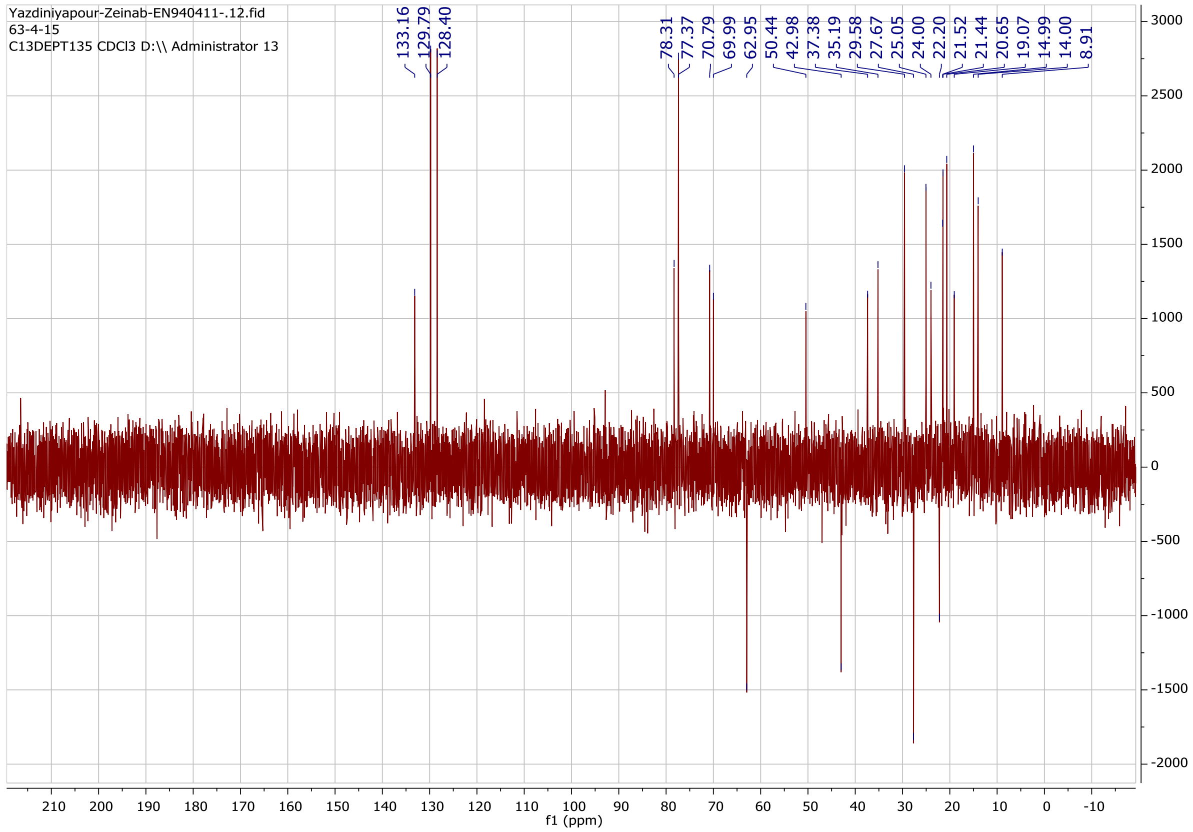

Figure S14. ^{13}C DEPT135 (100 MHz) spectrum of compound **2**

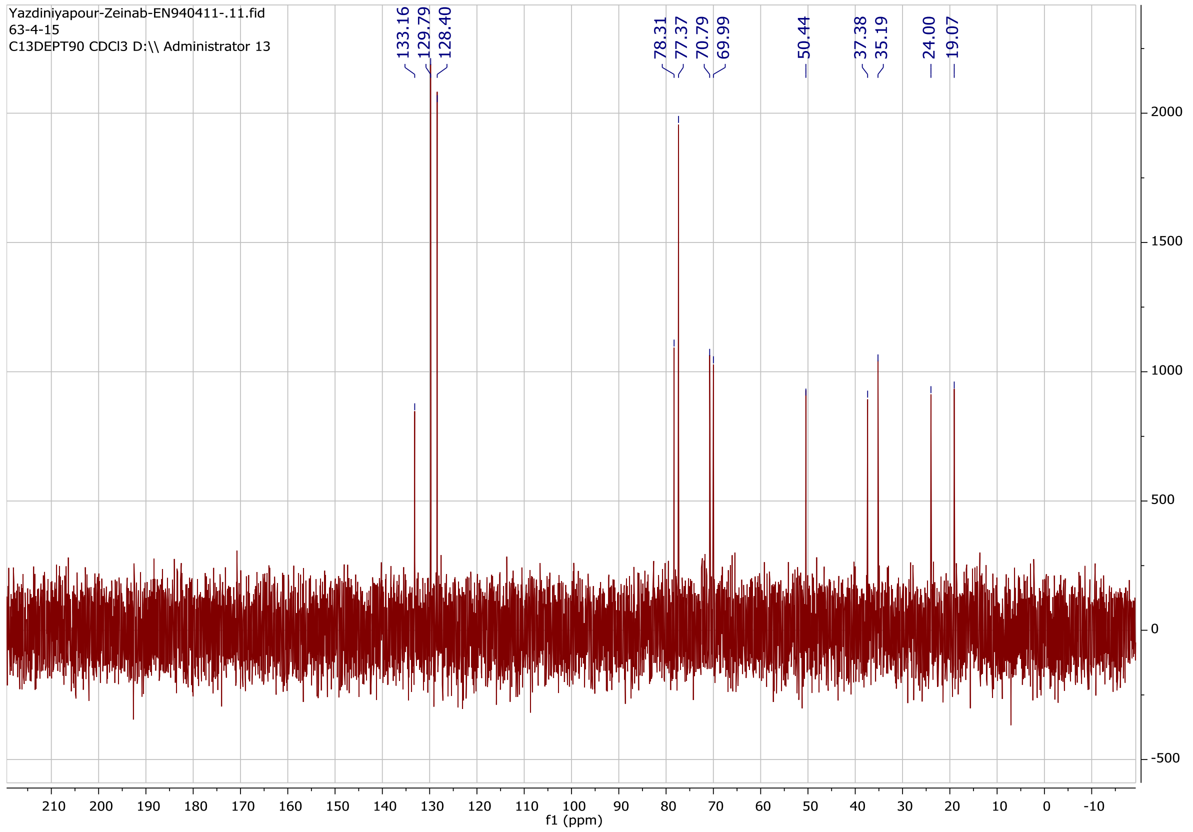

Figure S15. ^{13}C DEPT90 (100 MHz) spectrum of compound **2**

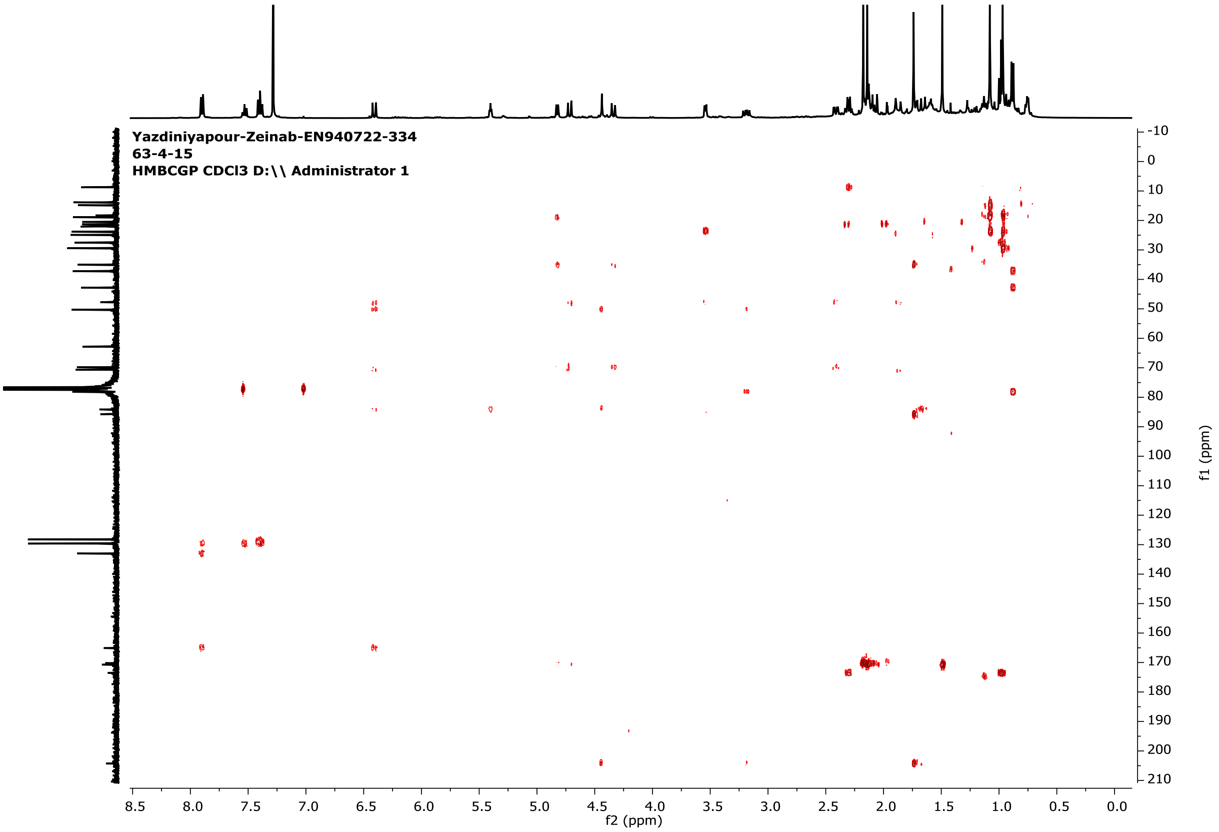

Figure S16. HMBCGP (400 MHz) spectrum of compound **2**

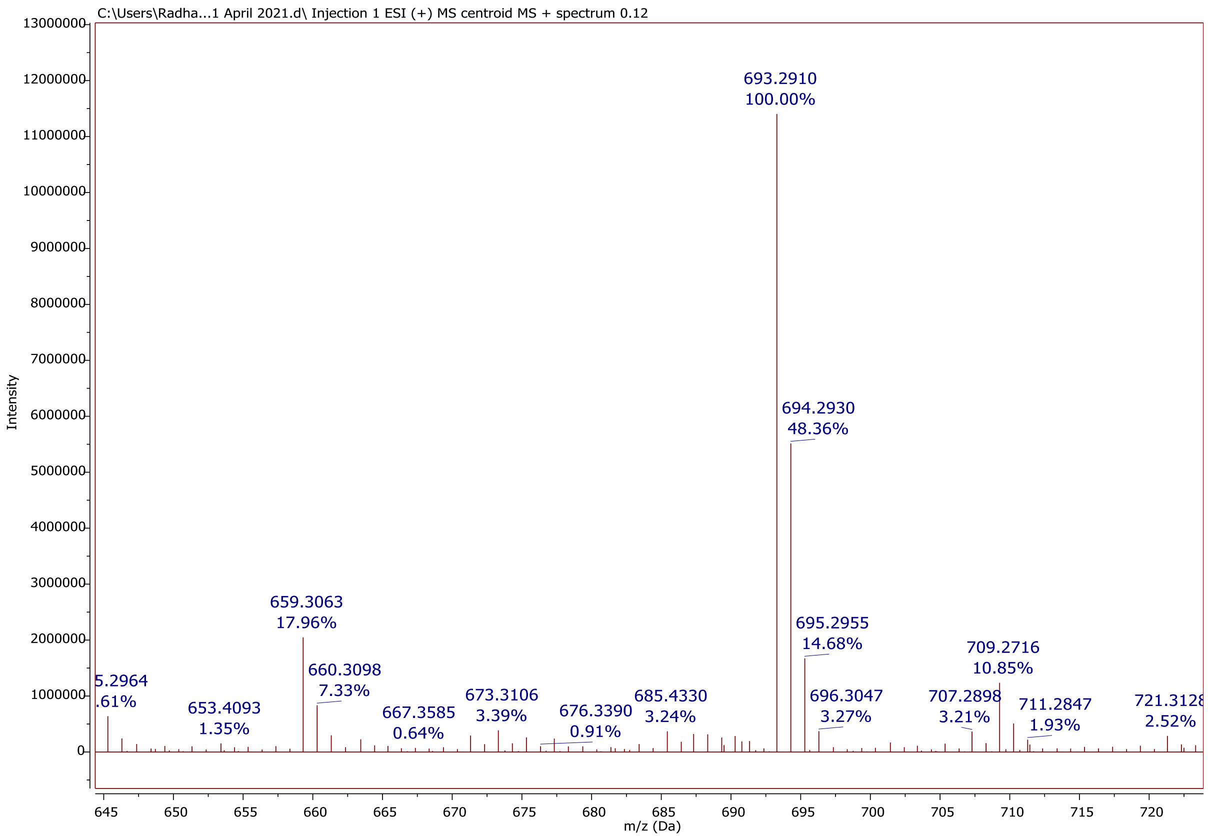

Figure S17. HR-ESI-MS spectrum of compound **2**

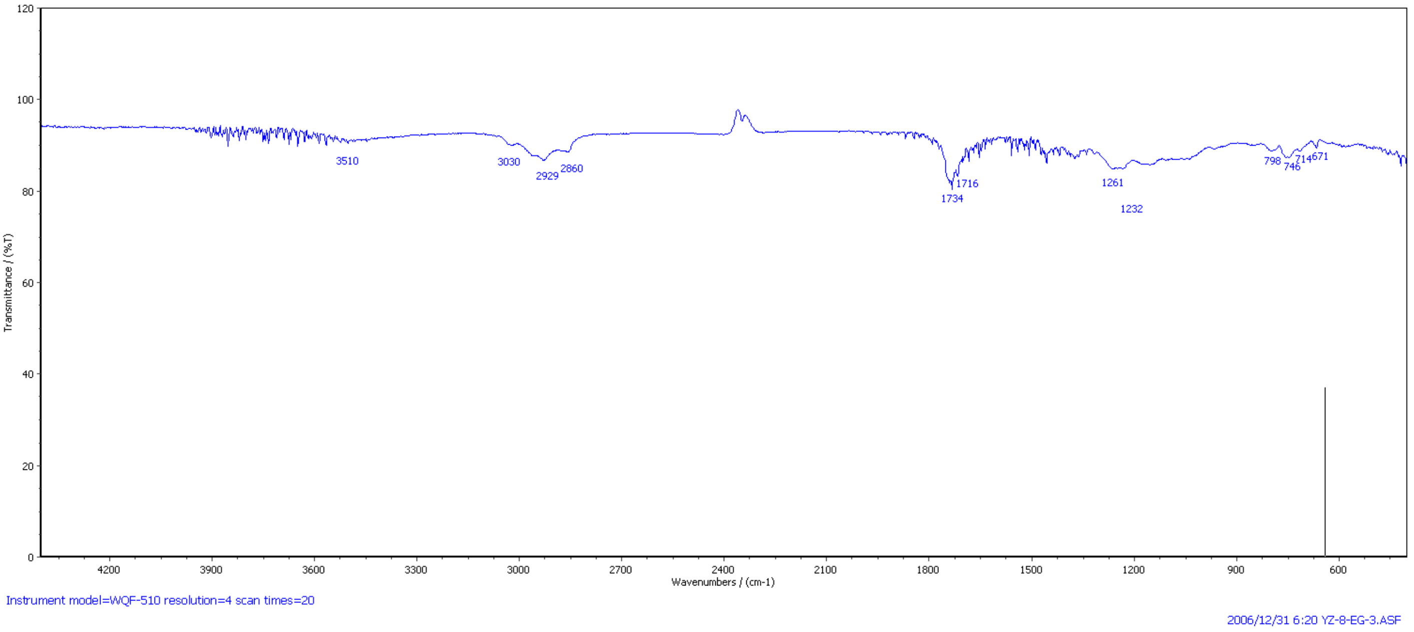

Figure S18. FT-IR spectrum of compound **2**

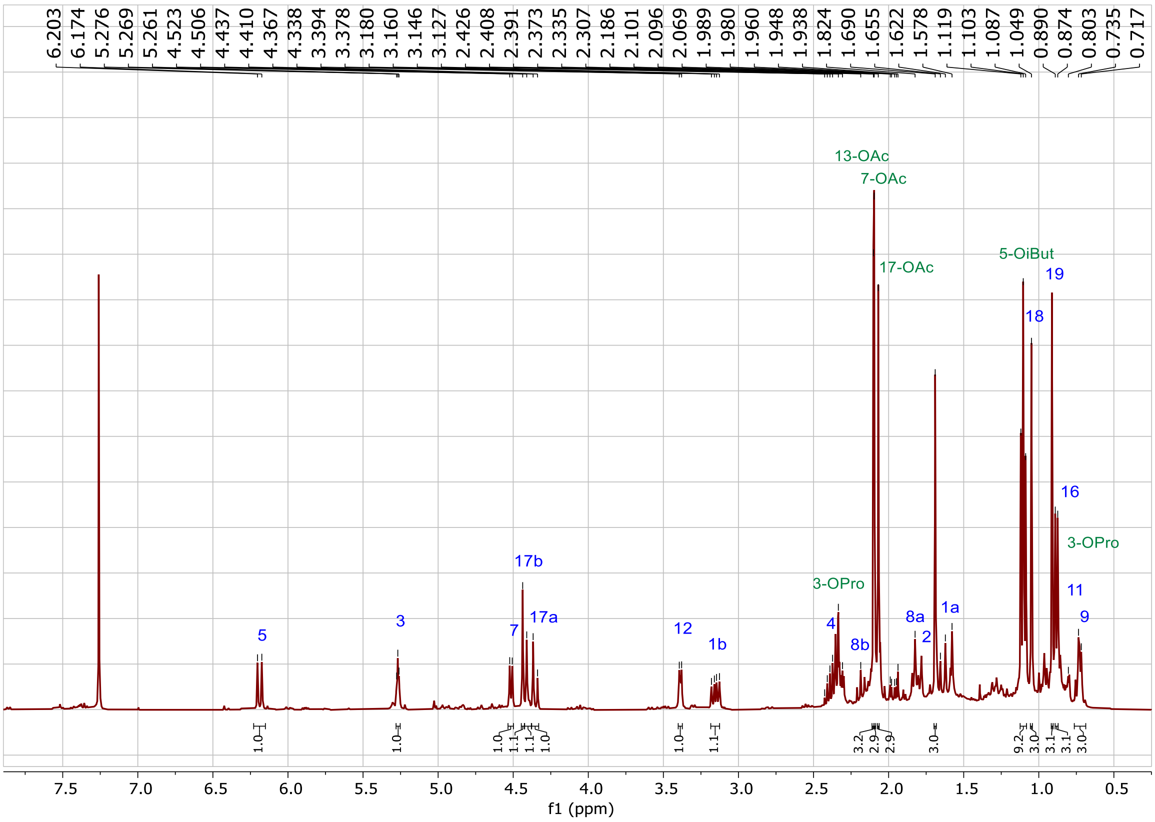

Figure S19. ^{1}H NMR (400 MHz) spectrum of compound **3**

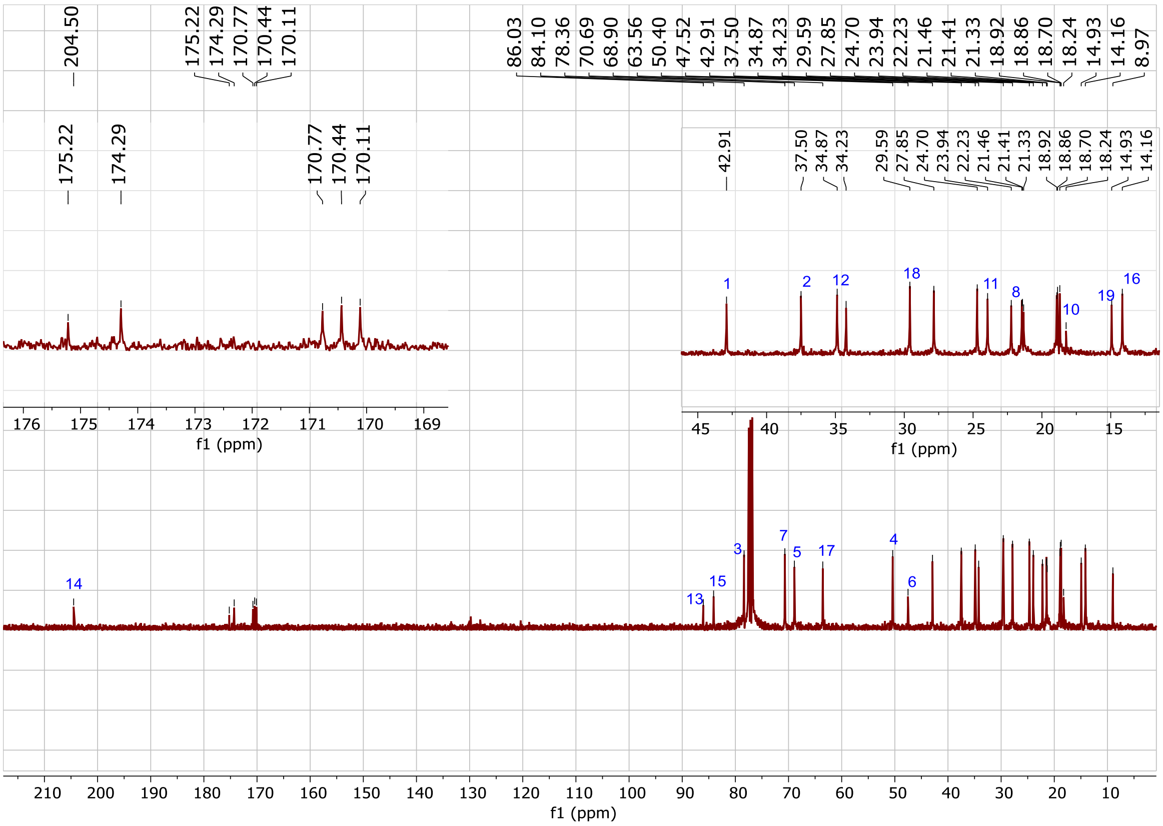

Figure S20. ^{13}C NMR (100 MHz) spectrum of compound **3**

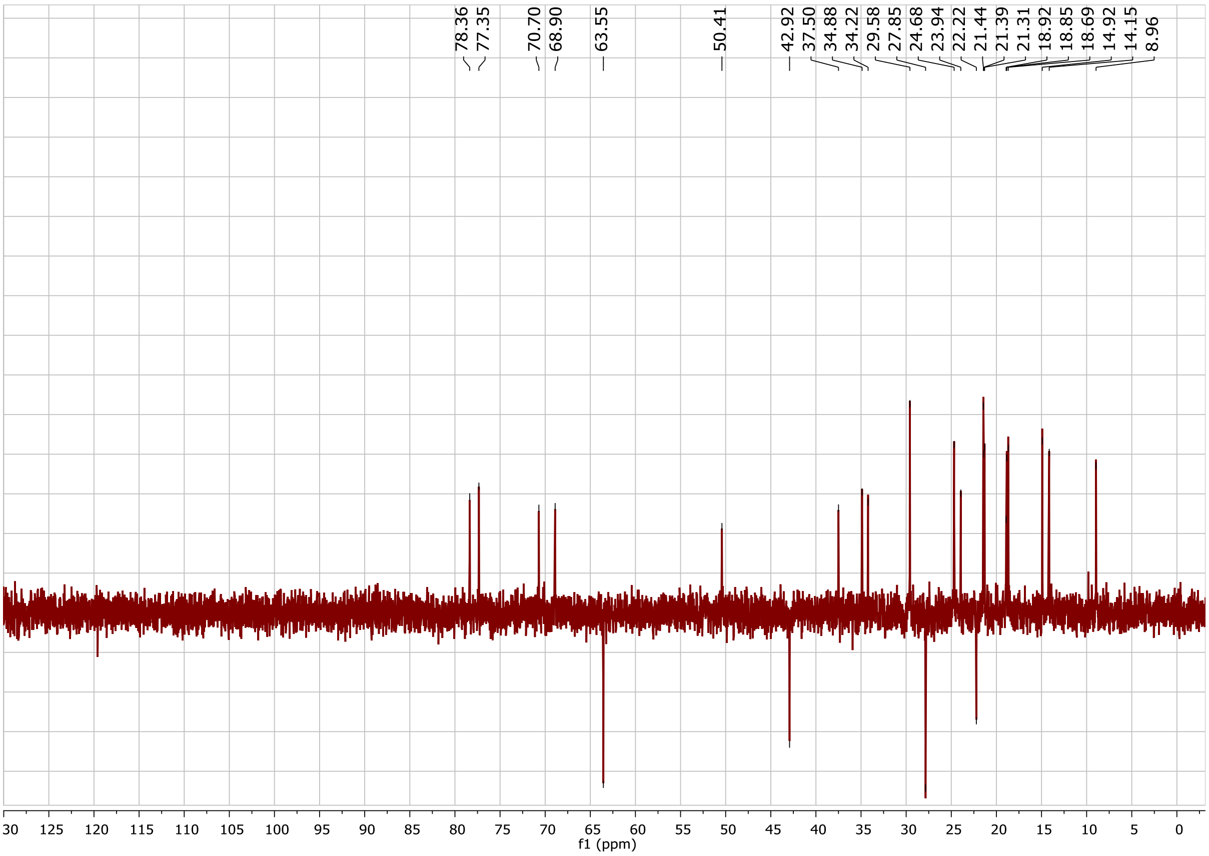

Figure S21. ^{13}C DEPT135 (100 MHz) spectrum of compound **3**

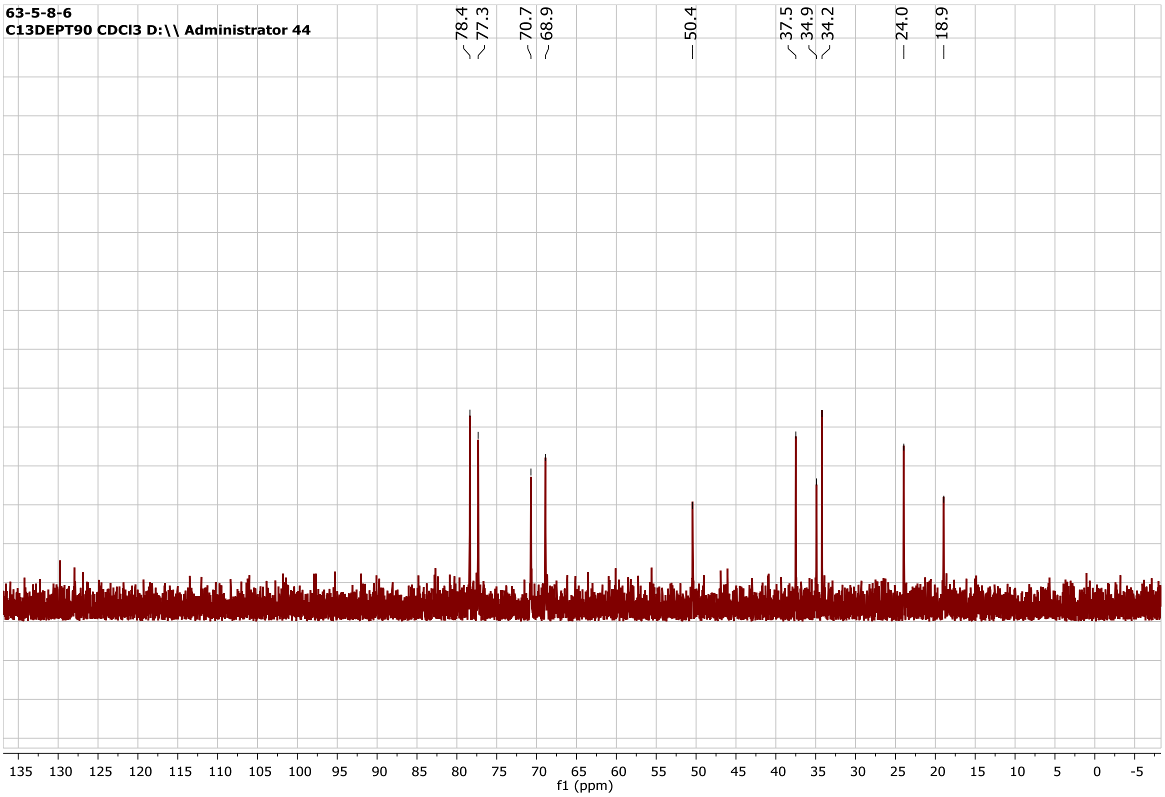

Figure S22. 13C DEPT90 (100 MHz) spectrum of compound **3**

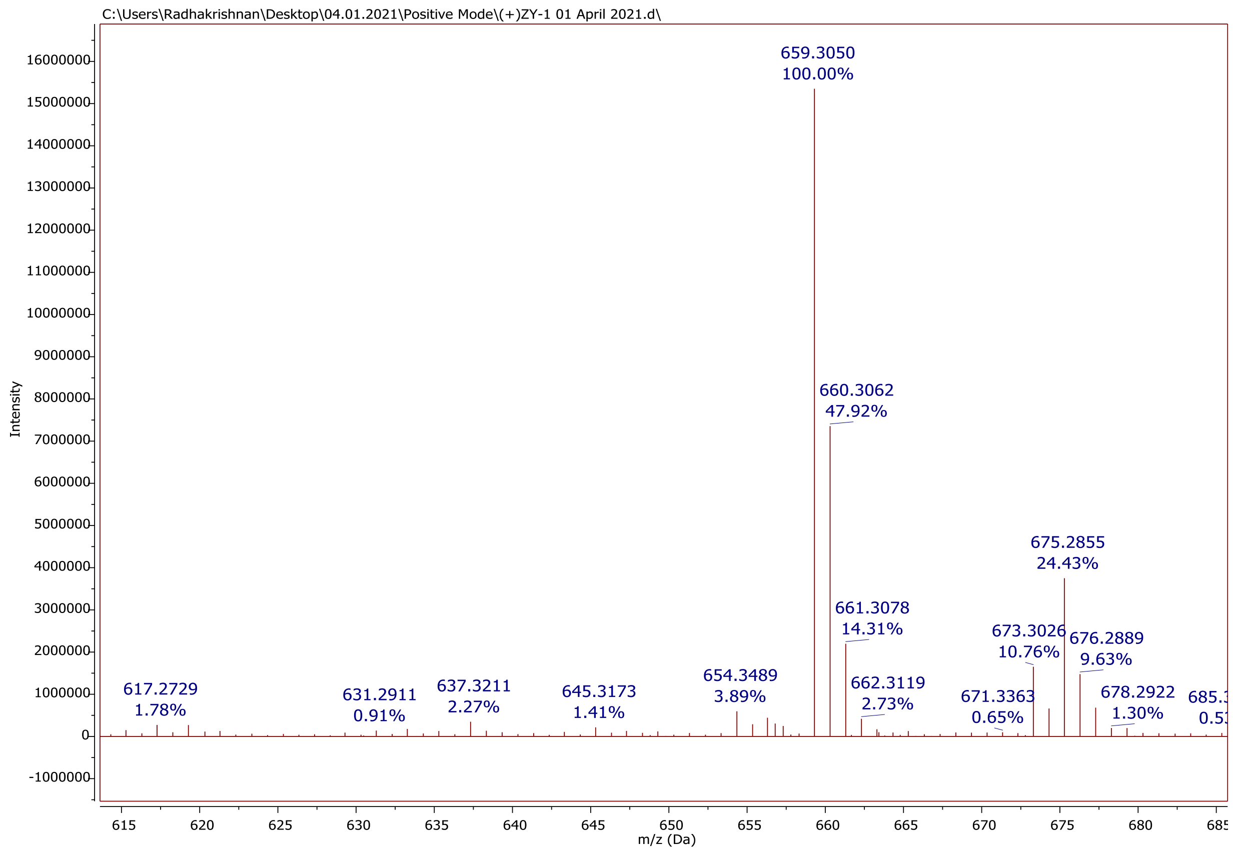

Figure S23. HR-ESI-MS spectrum of compound 3

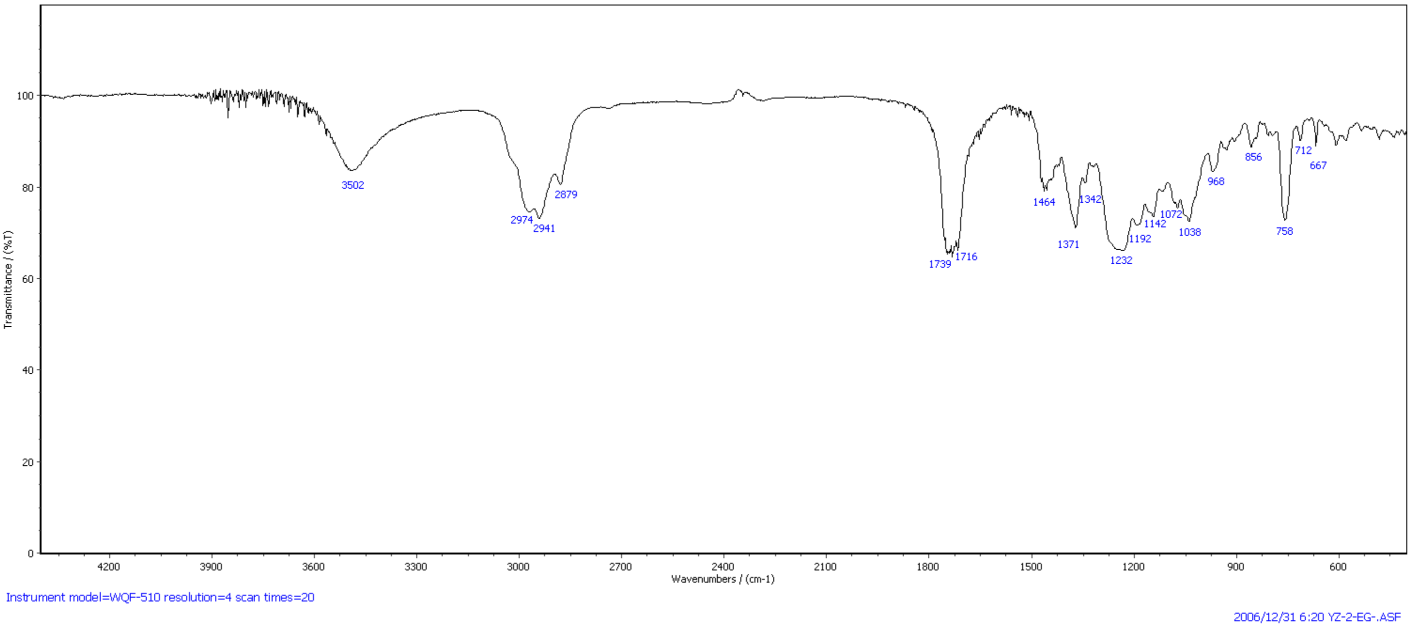

Figure S24. FT-IR spectrum of compound **3**

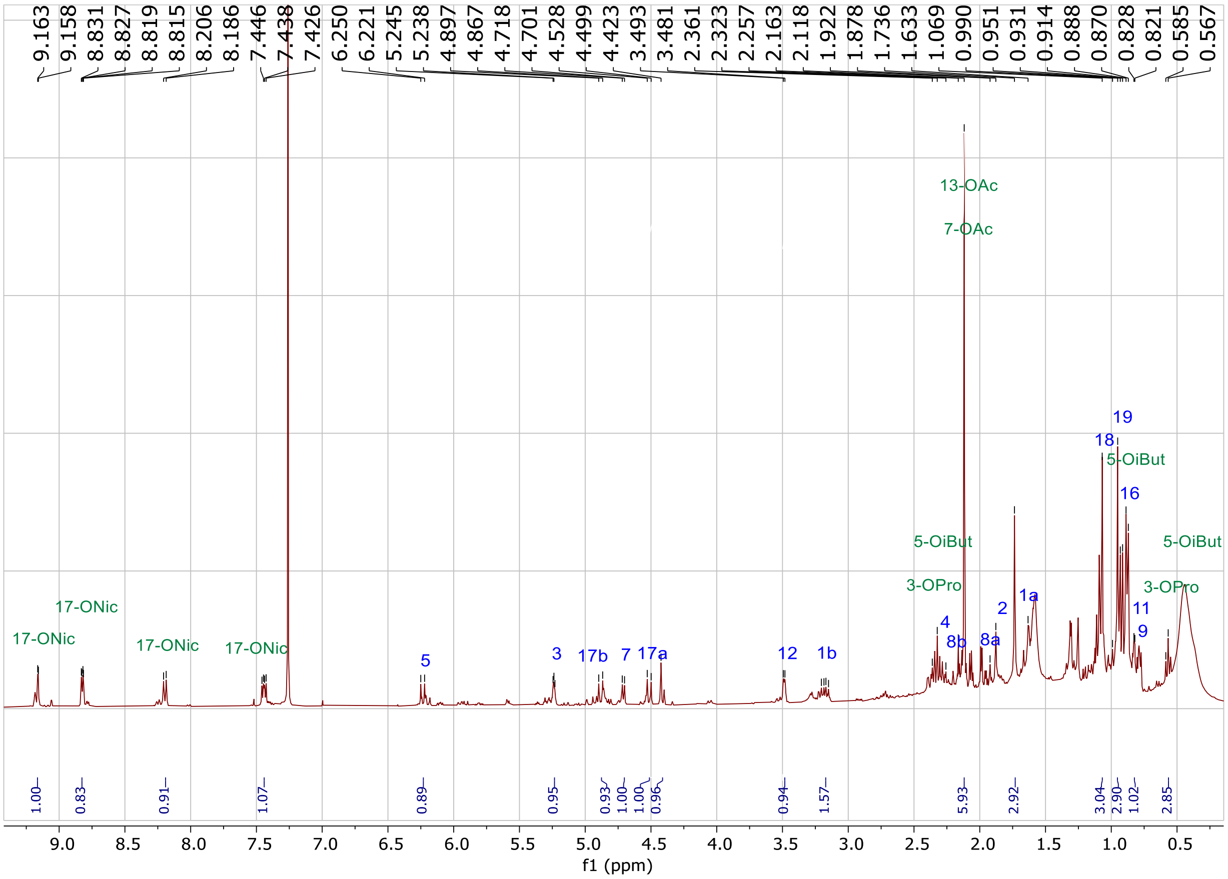

Figure S25. ^{1}H NMR (400 MHz) spectrum compound **4**

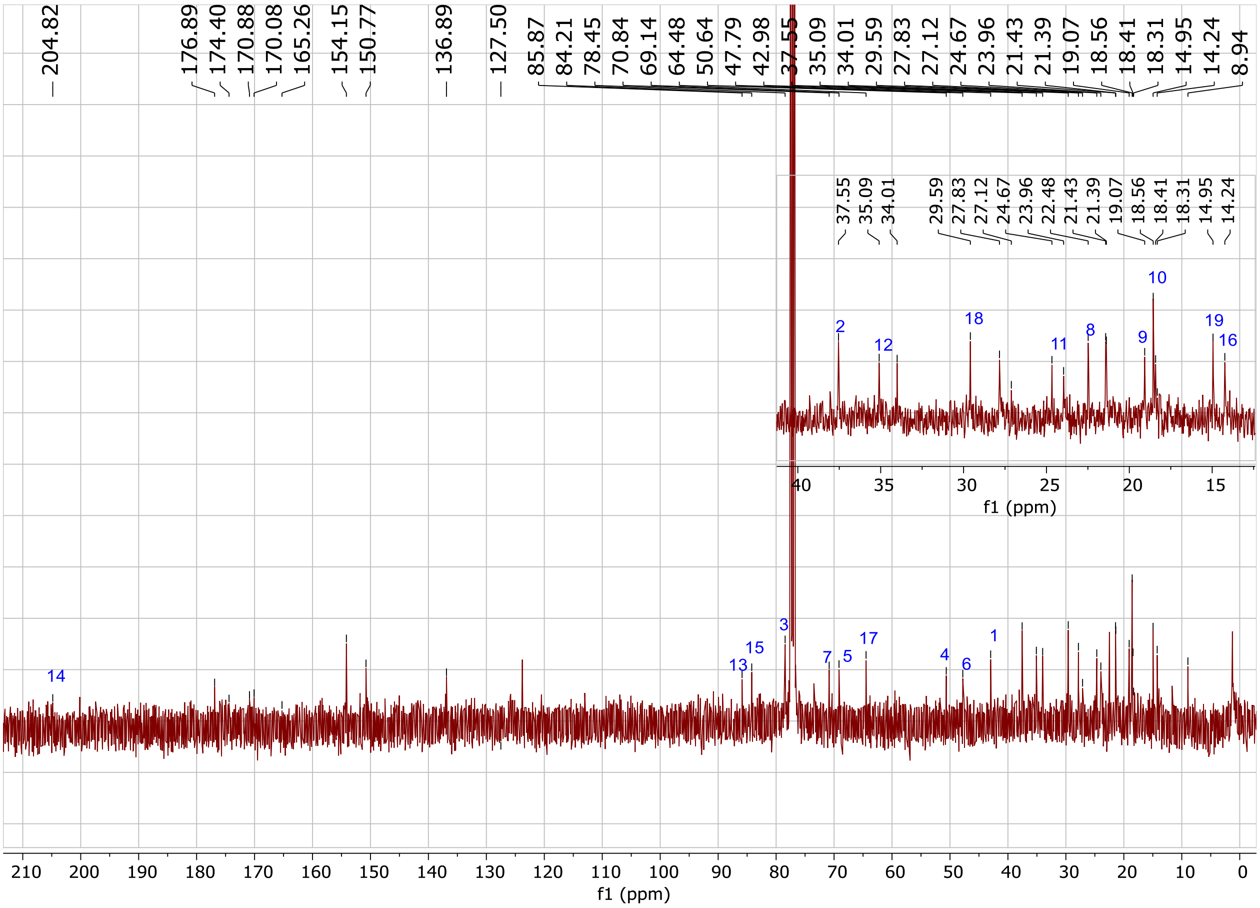

Figure S26. ^{13}C NMR (100 MHz) spectrum of compound 4

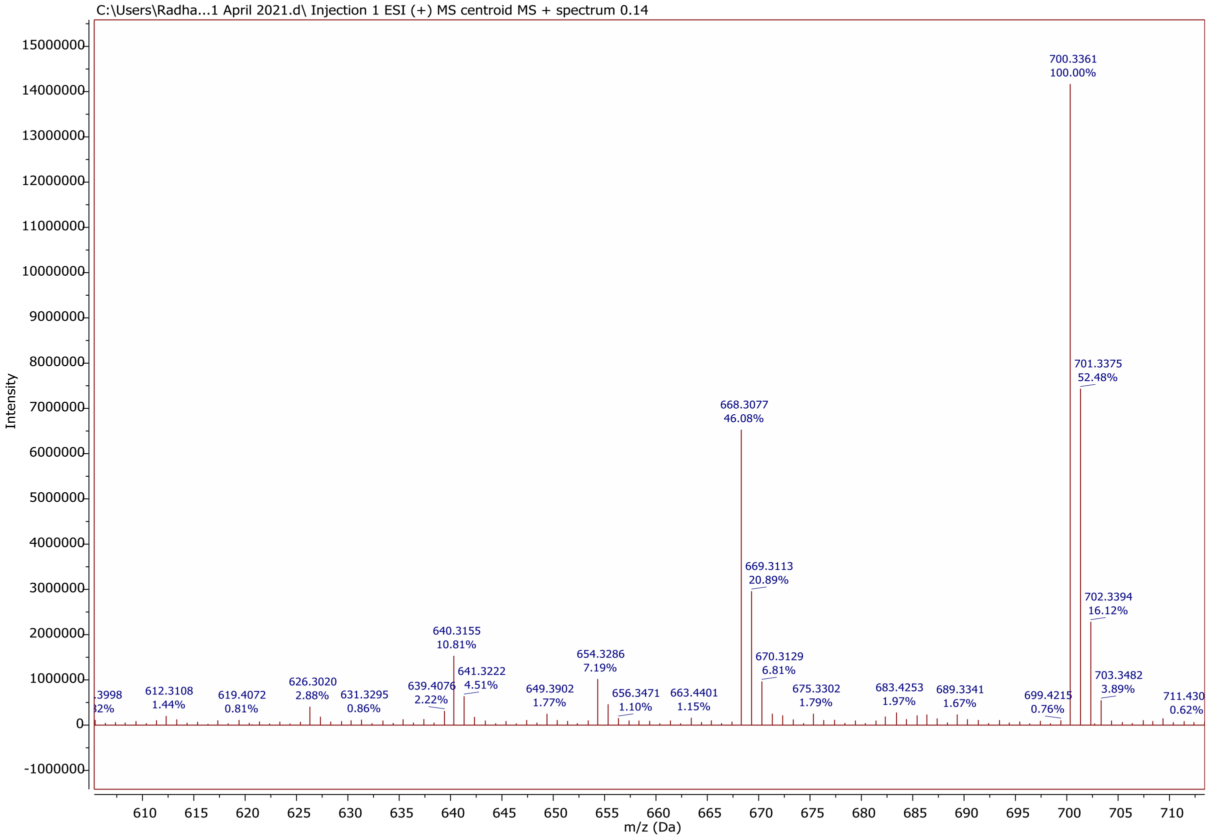

Figure S27. HR-ESI-MS spectrum of compound 4

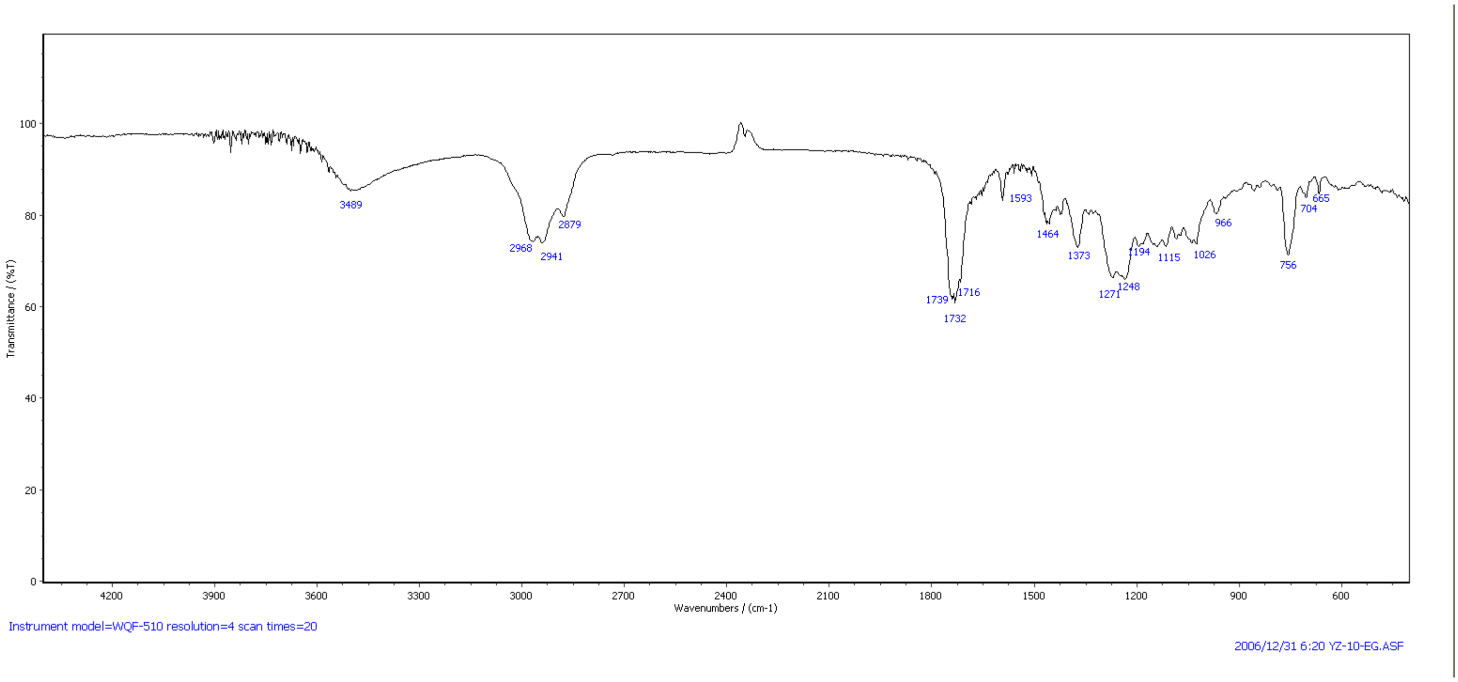

Figure S28. FT-IR spectrum of compound **4**

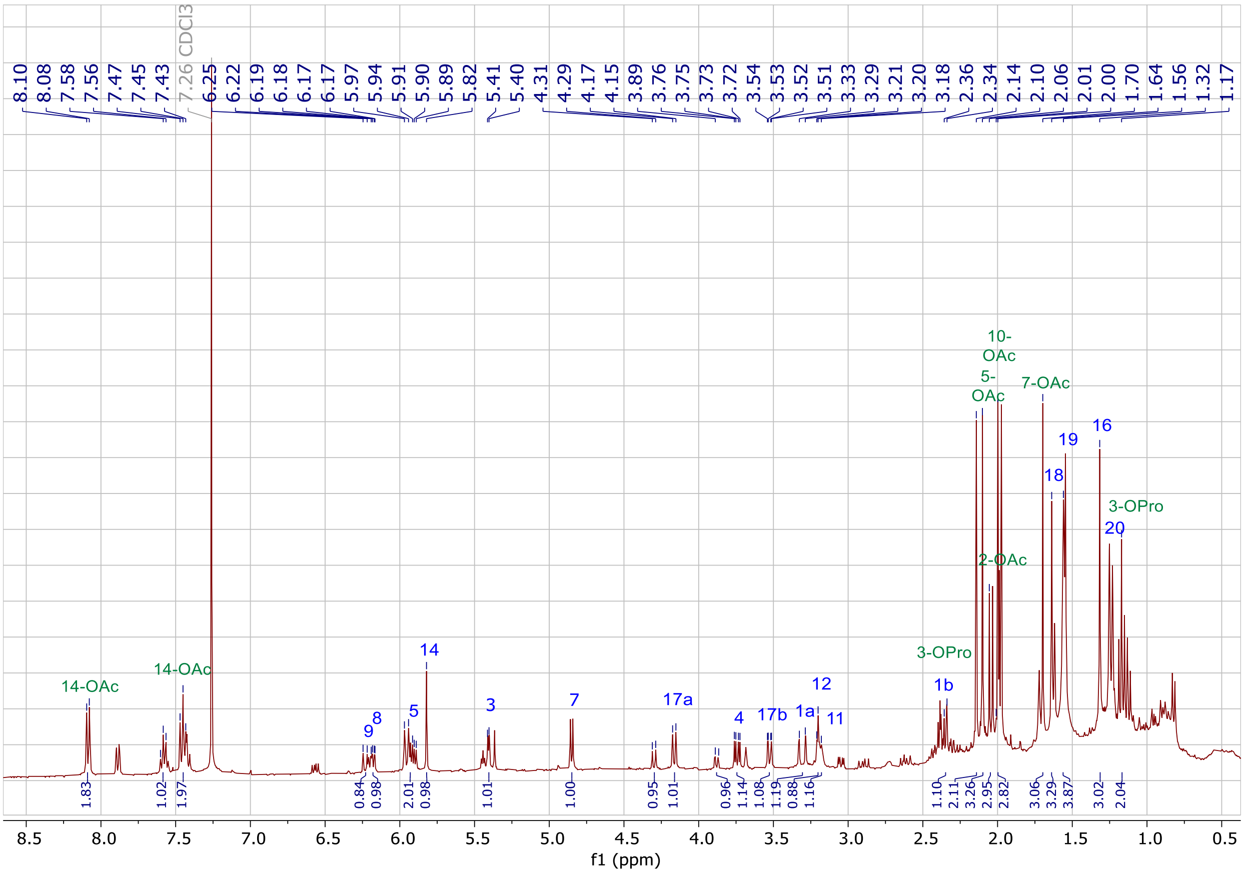

Figure S29. ^{1}H NMR (400 MHz) spectrum of compound **5**

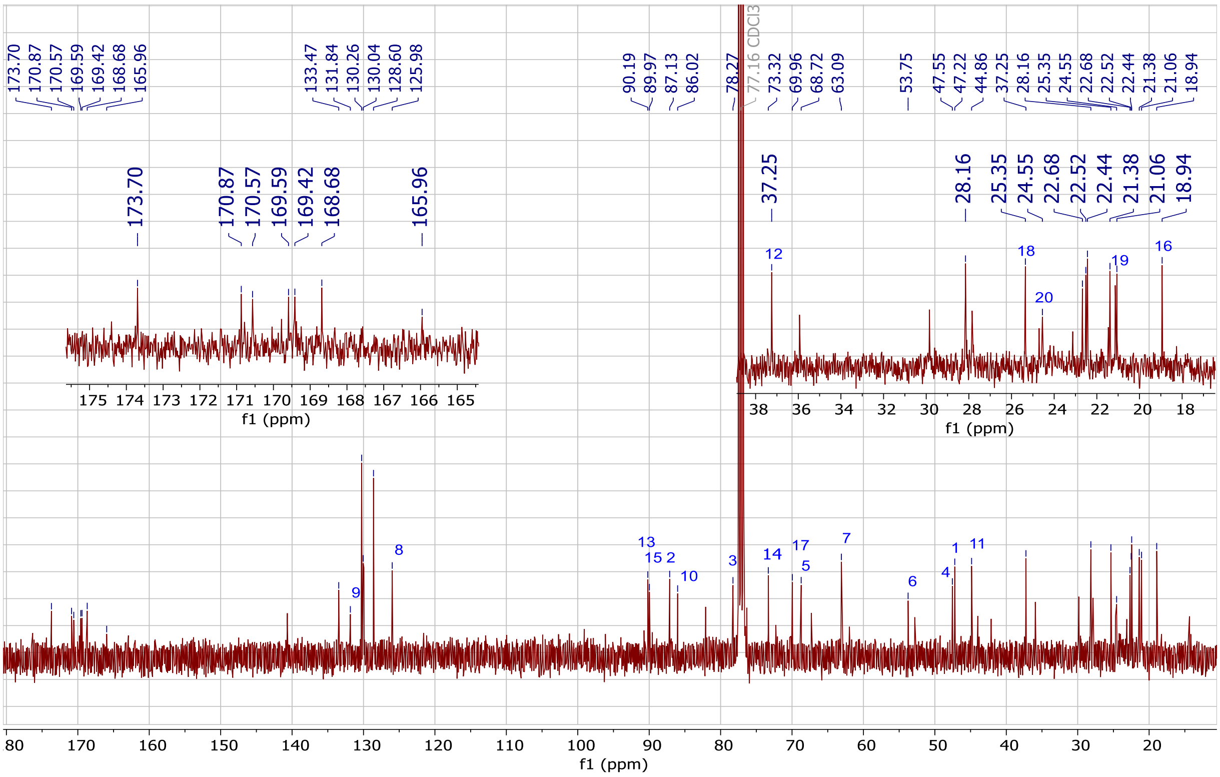

Figure S30. ^{13}C NMR (100 MHz) spectrum of compound **5**

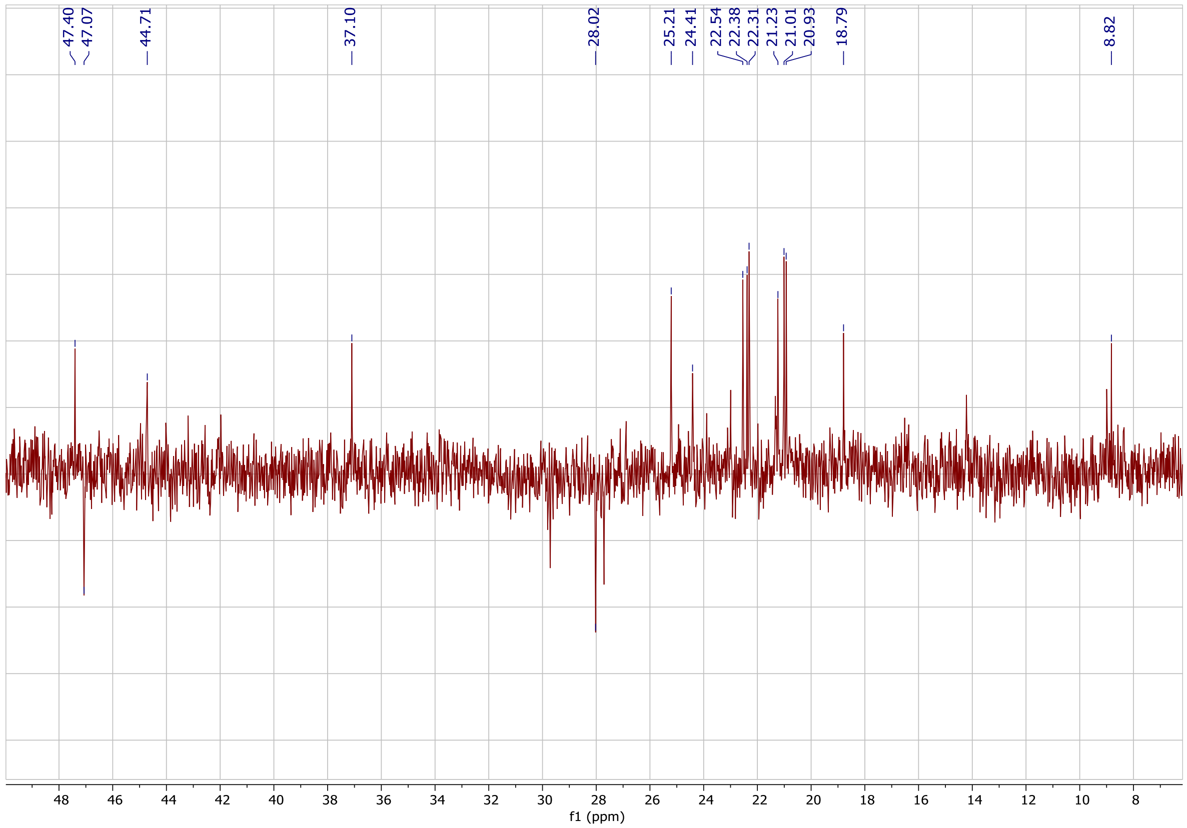

Figure S31. ^{13}C DEPT135 (100 MHz) spectrum of compound **5**

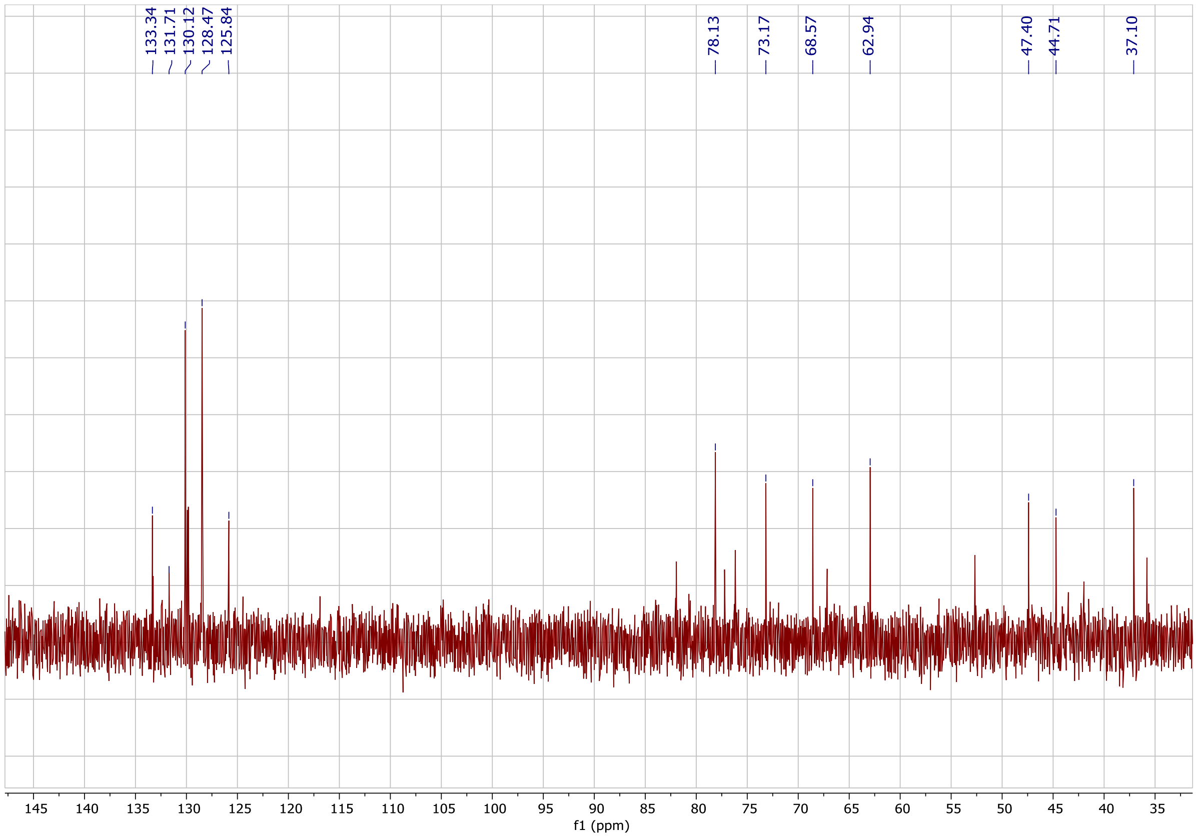

Figure S32. ^{13}C DEPT90 (100 MHz) spectrum of compound **5**

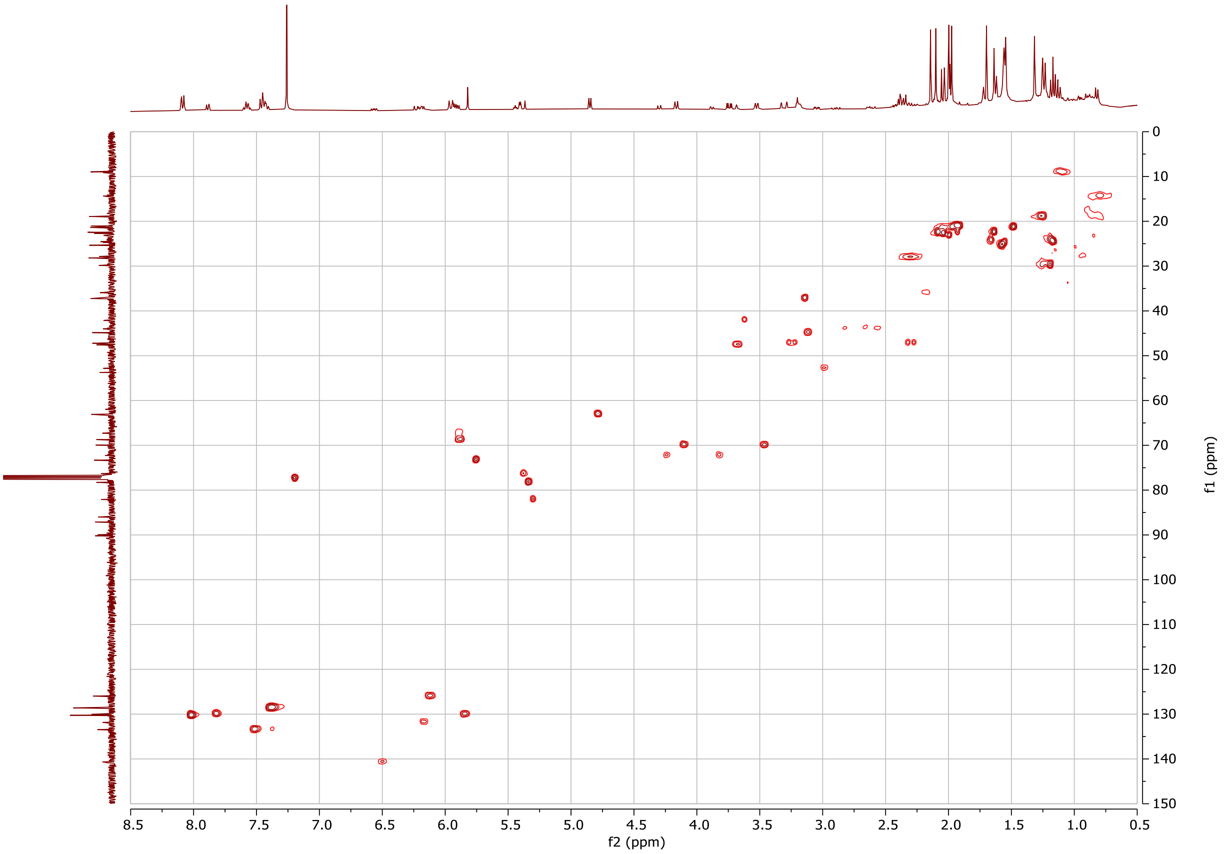

Figure S33. HSQCGP (400 MHz) spectrum of compound **5**

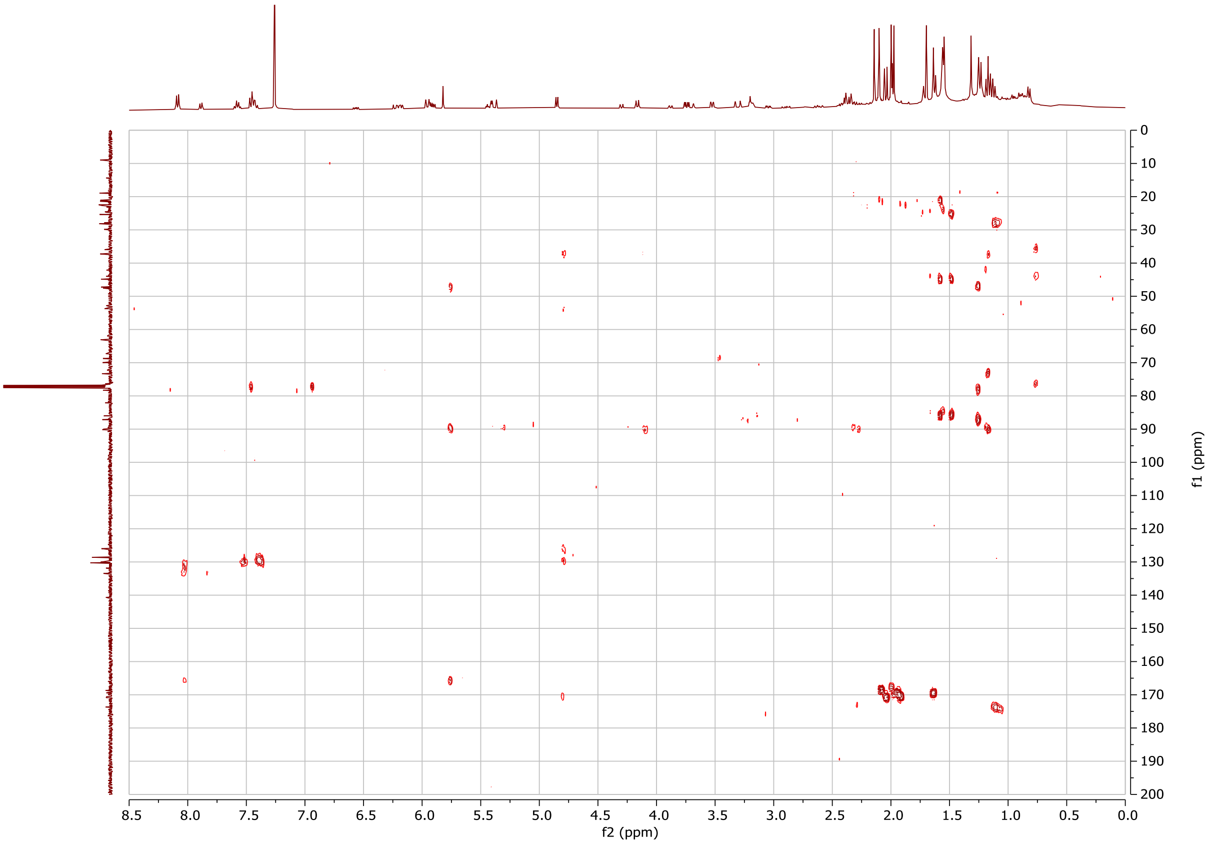

Figure S34. HMBCGP (400 MHz) spectrum of compound **5**

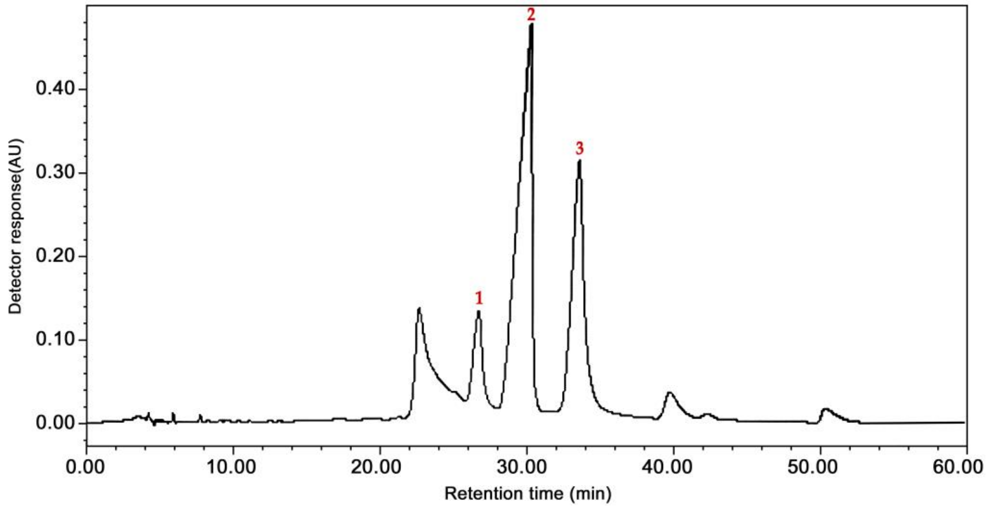

Figure S35. HPLC chromatogram of fraction **5** showing peaks corresponding to compounds **1, 2** and **3**

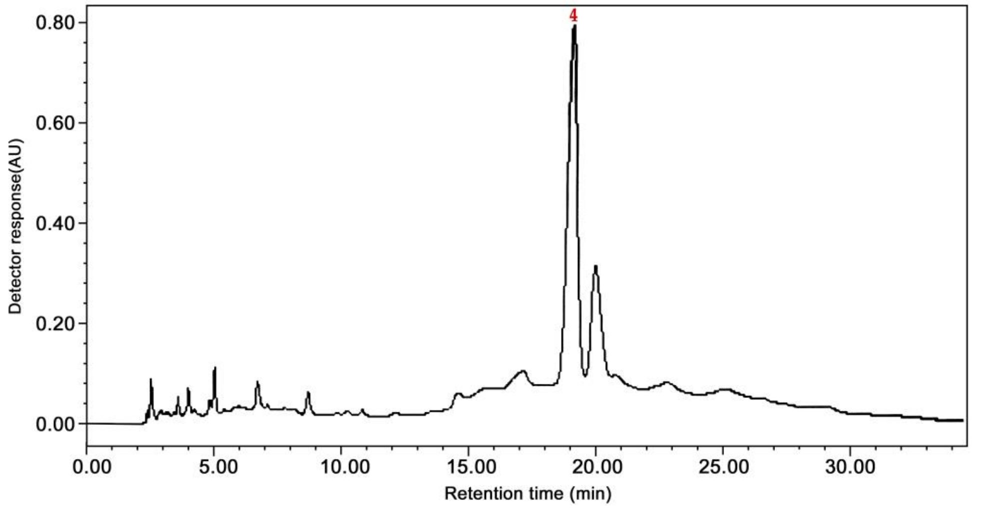

Figure S36. HPLC chromatogram of fraction **10-1** showing peak corresponding to compound **4**

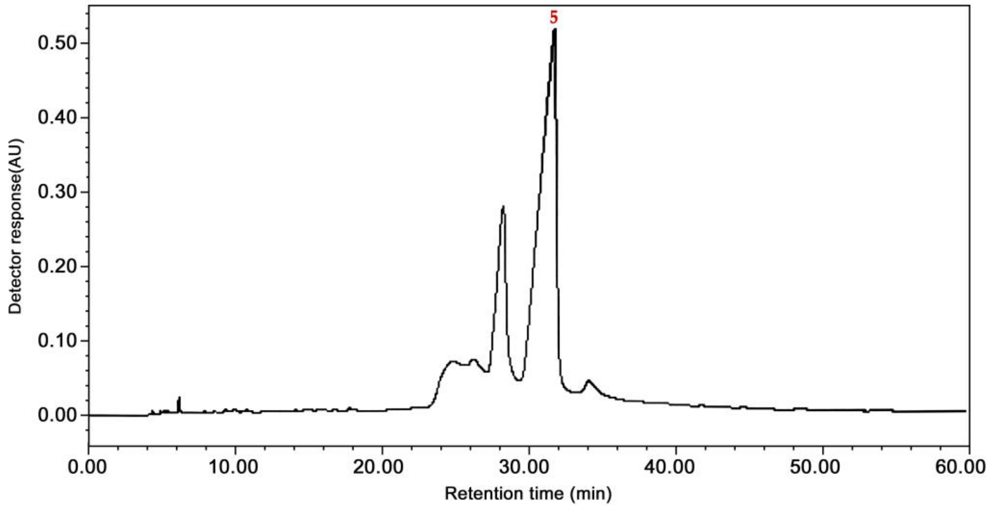

Figure S37. HPLC chromatogram of fraction **9** showing peak corresponding to compound **5**
